# Targeting STEC-induced edema disease in weaned piglets: prophylactic oral phage P-GXEC-L2P5 attenuates bacterial colonization, toxin production, and endothelial damage

**DOI:** 10.1186/s13567-025-01683-w

**Published:** 2025-12-17

**Authors:** Runwen Ma, Shuming Tang, Jialing Zeng, Yixian Wei, Xun Li, Xiaoye Wang

**Affiliations:** 1 College of Animal Science and Technology, Guangxi Key Laboratory of Animal Reproduction, Breeding and Disease Control, Guangxi Zhuang Autonomous Region Engineering Research Center of Veterinary Biologics, Guangxi University, Nanning, 530004 P. R. China; 2 HeBei Province Center for Animal Disease Prevention and Control, Shijiazhuang, 050000 P. R. China

**Keywords:** Bacteriophage, STEC, edema disease, prophylactic therapy

## Abstract

Edema disease (ED), a fatal disease in weaned piglets, is caused by Shiga toxin-producing *Escherichia coli* (STEC). The increasing emergence of antibiotic-resistant *E. coli* strains has necessitated the exploration of alternatives such as bacteriophage therapy. Using a porcine model, this study evaluated the prophylactic effect of bacteriophage P-GXEC-L2P5 administered by oral gavage against ED. The novel phage P-GXEC-L2P5 was isolated using a multidrug-resistant (MDR) STEC strain GXEC-STL2 as the host. P-GXEC-L2P5 was identified as a member of *Caudoviricetes*, *Dhillonvirus*, with an 88,607 base pair (bp) genome, and it possessed a short latent period (10 min), moderate pH stability (5–10), and appropriate thermal tolerance (4–60 ℃). Piglets pretreated with P-GXEC-L2P5 showed no apparent clinical signs (e.g., eyelid edema or neurological symptoms) after challenge with GXEC-STL2. B-scan ultrasound revealed no significant hydronephrosis. Necropsy showed only mild intestinal congestion, with no other gross pathological lesions noted. Histopathology demonstrated no significant differences in features compared with noninfected controls. Phage treatment significantly reduced fecal STEC shedding (*P* < 0.05) and significantly decreased Stx2e concentrations in serum, cerebral cortex, kidney, and small intestine (*P* < 0.01). The messenger RNA (mRNA) expression of Gb4 (Stx2e receptor) was significantly lower in these tissues (*P* < 0.05). Concurrently, vascular endothelial cells exhibited increased FITC-labeled wheat germ agglutinin (FITC-WGA) fluorescence intensity and increased mRNA expression of endothelial integrity factors (connexin43, vinculin, and zonula occludens-1; *P* < 0.05). In addition, phage treatment preserved jejunal microbiota diversity and abundance. In conclusion, P-GXEC-L2P5 effectively prevented STEC-induced ED by reducing STEC load and Stx2e levels, while mitigating increased vascular permeability.

**Supplementary Information:**

The online version contains supplementary material available at 10.1186/s13567-025-01683-w.

## Introduction

Edema disease (ED) is a fatal vascular disease in weaned piglets caused by Shiga toxin-producing *Escherichia coli* (STEC) [1], with mortality rates exceeding 50% in untreated weaned piglets [2]. The pathogenesis hinges on Shiga toxin 2e (Stx2e), which binds to receptor globotetraosylceramide (Gb4) on vascular endothelium, increases vascular permeability, and eventually triggers systemic edema and multiorgan failure [3].

Conventional therapies, including broad-spectrum antibiotics, face mounting challenges owing to the rampant emergence of multidrug-resistant *E. coli* strains [4, 5]. Currently, a variety of novel therapeutic approaches are under active investigation, and bacteriophages are among these approaches [6].

Bacteriophage therapy has a long history [7, 8]. As viruses that kill bacteria, bacteriophages have had their therapeutic value in treating bacterial infections reassessed over the last 20 years [9]. Lytic phages are exclusively utilized in therapeutic applications [10]. These phages inhibit the development of antimicrobial resistance (AMR) by specifically lysing host bacteria while preserving the microbiota more effectively than broad-spectrum antibiotics [11]. There is a growing body of research examining the efficacy of phage treatments in swine [12, 13]. Mao et al. [14] and Li et al. [15] demonstrated that phage therapy could alleviate the severity of clinical symptoms in piglets infected with *Escherichia coli*. Not only did phage therapy reduce pathological lesions in intestinal tissues, but it also had no significant impact on the relative abundance of beneficial bacteria in the gut microbiota of piglets, while the number of pathogenic bacteria was significantly reduced. In a study conducted in 2022, Kim et al. [16] evaluated the effect of phage EK99P-1 against ETEC K99-infected the porcine intestinal epithelial cell line (IPEC-J2). Phage EK99P-1 prevented ETEC K99-induced barrier disruption by attenuating the increased permeability mediated by the loss of tight junction proteins such as zonula occludens-1 (ZO-1), occludin, and claudin-3, suggesting that phage EK99P-1 prevented ETEC K99-induced barrier dysfunction in IPEC-J2 cells and alleviated inflammation caused by ETEC K99 infection. Furthermore, under practical production conditions, Zeng et al. [17] demonstrated that dietary supplementation with a bacteriophage cocktail (0.4 g/kg diet, 1 × 10^6^ PFU/g) significantly increased final body weight, average daily feed intake, average daily gain, and gain-to-feed ratio in weaned piglets. However, data on phage applications against swine-specific STEC pathotypes, particularly those producing Stx2e, remain limited in piglet models.

In this study, we investigated the prophylactic efficacy of phage P-GXEC-L2P5 administered by oral gavage in the porcine ED model and systematically assessed its capacity to reduce bacterial colonization, toxin dissemination, and endothelial damage during ED pathogenesis.

## Materials and methods

### Bacterial isolation and identification

GXEC-STL2 was used for the challenge experiments. This strain was isolated from the feces of piglets suffering from diarrhea and neurological symptoms at a porcine farm in Nanning, China. The bacterial isolation and identification methods were employed according to established protocols [15, 18]. In addition, its DNA was extracted using the TIANamp Bacteria DNA Kit (LOT: A0806A, TIANGEN, China), and DNA sequencing was subsequently conducted by Sangon Biotech Co., Ltd. (Shanghai, China). Virulence genes were amplified via polymerase chain reaction (PCR) using the primers listed in Additional file 1, and antibiotic susceptibility was determined using the Kirby–Bauer (K–B) disk diffusion method as described by the Clinical and Laboratory Standards Institute (CLSI, 2019).

### Phage isolation and characterization

On the basis of previous experimental methods [18–20], phage P-GXEC-L2P5 (GenBank: PQ843332.1) was isolated from the sewage of a porcine farm in Nanning, China, using GXEC-STL2 as the host bacterium. Subsequently, its host range, morphological characteristics, optimal multiplicity of infection (MOI), one-step growth kinetics, thermal and pH stability, and in vitro antibacterial activity were characterized; in addition, genomic extraction and analysis were performed.

### Animals and animal treatment

Eighteen 21-day-old piglets were purchased from a group company’s sow farm in Guilin, China, and the experiment was initiated after 1 day of acclimatization. Subsequently, these animals were randomly divided into three groups: negative group (NG, *n* = 6): each piglet received 20 mL reconstituted milk (RM) (new formula of Opticare Milk, Swinco International, the Netherlands), followed by an additional 20 mL RM 2 hours later. Positive group (PG, *n* = 6): each piglet received 20 mL RM, followed by 10 mL GXEC-STL2 (1 × 10^8^ CFU/mL) mixed with 10 mL RM 2 hours later. Phage prophylaxis group (PPG, *n* = 6): each piglet received 10 mL P-GXEC-L2P5 (1 × 10^9^ PFU/mL) mixed with 10 mL RM, followed by 10 mL GXEC-STL2 (1 × 10^8^ CFU/mL) mixed with 10 mL RM 2 hours later. The procedure was repeated daily for 7 days in each group. All groups were allowed free access to feed and water, and no antibiotics were used for treatment throughout the experiment. The first day of treatment was designated as day 1, and all piglets were euthanized on day 14 for sample collection.

### Clinical evaluation

Guided by previous veterinary studies [21], we developed a standardized clinical grading framework for disease assessment (Additional file 2). Piglets in each group were clinically observed and assessed daily. Necropsies were performed on all piglets on day 14.

### B-scan ultrasonography in the kidneys

B-scan ultrasound imaging of the kidneys was performed in each group of piglets on days 0, 7, and 14. To ensure diagnostic objectivity and reliability, the sonographer was blinded to group allocations.

### Histopathological analysis

Tissue samples were fixed in 4% paraformaldehyde for ≥ 24 h and then processed through graded ethanol dehydration and paraffin embedding. We prepared 4-µm-thick sections and stained them with hematoxylin and eosin (H&E) for histopathological examination using light microscopy.

### Detection of STEC load in feces

Fecal samples were collected from piglets in each group on days 0, 1, 4, 7, and 14. As described in previous studies, total DNA was extracted from fecal samples using the standard phenol-chloroform method [22]. The Stx2e primer sequences were as follows: Stx2e-F: 5′-GCCCGGTGTGACAACTATT-3′, Stx2e-R: 5′-CCAGTGAGTGACTGATTT-3′, Stx2e probe: 5′-CCATGACAACGGACAGCAGTTATACCA-3′. A total of 20 μL reaction system was prepared for amplification with 10 μL 2 × Green Taq PCR Master Mix (Nanjing Vazyme Biotech Co., Ltd, China), 6.6 μL ddH₂O, 0.4 μL probe (10 μM), 1 μL each of forward and reverse primers (10 μM), and 1 ng template DNA. Fecal STEC load was quantified on the basis of Ct values and a standard curve generated via quantitative real-time PCR (qPCR) using bacterial samples of known concentrations.

### Detection of STEC Stx2e in serum and tissues

Serum samples were collected from piglets in each group on days 0, 1, 4, 7, and 14, and duodenal, jejunal, ileal, as well as cerebral cortical and renal tissue samples were collected on day 14. The concentrations of Stx2e in serum and tissues were quantified according to the operating procedures of the Porcine *Escherichia coli* Stx2e Enzyme Immunoassay Kit (Jiangsu Enzyme Exemption Industry Co., Ltd., China).

### FITC-labeled wheat germ agglutinin (FITC-WGA) staining

Tissue sections were deparaffinized, rinsed with distilled water, and subsequently washed twice (each for 5 min) in phosphate-buffered saline (PBS) containing 1% bovine serum albumin. Subsequently, the sections were incubated with FITC-WGA (Sigma-Aldrich, MO, USA) diluted in PBS in a humidified chamber at room temperature for 1 h. Afterward, the sections were washed three times with PBS (each for 5 min), mounted with a water-soluble mounting medium, and observed under a fluorescence microscope at Ex 495 nm/Em 515 nm to capture green fluorescence images.

### Gene expression of connexin43 (Cx43), vinculin (VCL), zonula occludens-1 (ZO-1), and globotetraosylceramide (Gb4)

The tissue samples were initially lysed and pretreated using Vazyme Trizol Extraction Kit (Nanjing, China) to obtain the tissue supernatant, after which total RNA was extracted using Axyprep Body Fluid Viral DNA/RNA Miniprep Kit (LOT: 21318KC5, CORNING, China). Qualified RNA samples were reverse-transcribed into complementary DNA (cDNA) using the Vazyme Reverse Transcription Kit (Nanjing, China), following the kit’s standardized protocol. Primers targeting Cx43, VCL, ZO-1, and Gb4 are shown in Additional file 3. The total volume of the qPCR reaction system was 20 µL, comprising the following components: 10 µL 2 × SYBR qPCR Master Mix (Nanjing Vazyme Biotech Co., Ltd., China), 0.4 µL forward primer (10 µM), 0.4 µL reverse primer (10 µM), 1 µL cDNA template, and 8.2 µL ddH₂O. The relative mRNA expression levels were calculated using the 2^−ΔΔCt^ method.

### Microbiota

Jejunal content samples were submitted to Novogene Bioinformatics Technology Co., Ltd. (Beijing, China) for 16S ribosomal RNA (rRNA) gene amplicon sequencing; subsequent bioinformatics analysis was performed on the QIIME2 platform. Bioinformatics analysis commenced with quality control of raw data and was followed by sequence merging, quality filtering, and chimera removal to generate the final high-quality dataset. Subsequent analyses included taxonomic annotation, microbial abundance profiling, and α-diversity/β-diversity assessments to compare jejunal microbiota across treatment groups.

### Statistical analysis

Data visualization was performed using GraphPad Prism 8.0.2 (GraphPad Software, Inc., San Diego, CA, USA). Statistical analyses were conducted using one-way analysis of variance (ANOVA) and Kruskal–Wallis tests in SPSS 23.0 (IBM SPSS, Chicago, IL, USA), with appropriate post hoc tests (e.g., Tukey’s test for one-way ANOVA) for multiple comparisons. Error bars indicate the mean ± standard deviation (SD). All experiments were conducted in triplicate. Statistical significance was set at *P* < 0.05.

## Results

### Biological characterization and genomic analysis of GXEC-STL2

**Figure 1:**
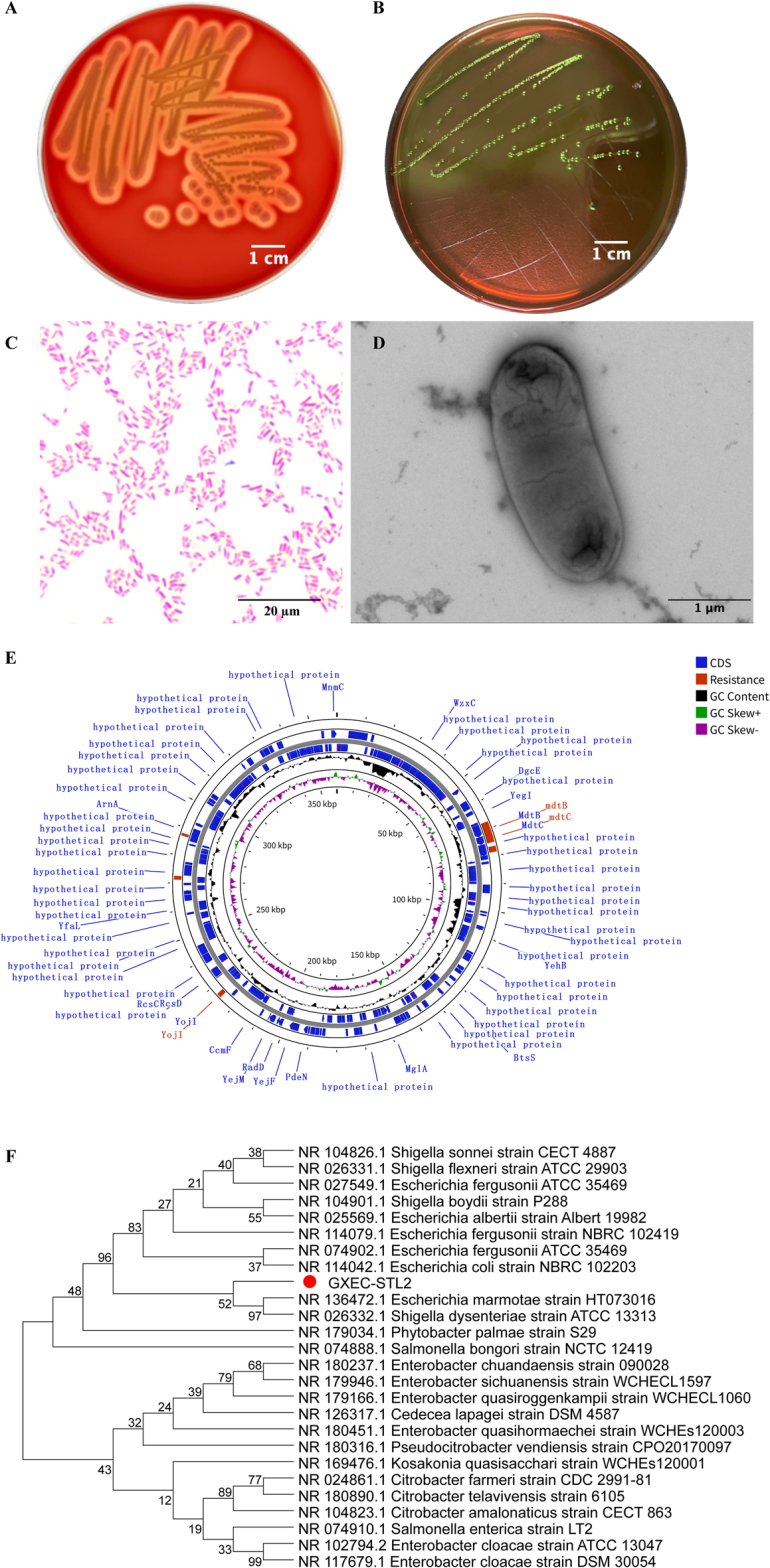
**Biological characteristics and genomic analysis of GXEC-STL2. A** Colony morphology on Columbia blood agar. Scale bar, 1 cm. **B** Colony morphology on EMD agar. **C** Gram-stained. Scale bar, 20 μm. **D** Transmission electron micrograph. Scale bar, 1 μm. **E** Comparative circular genome map. CDS, positive and negative GC, and resistance gene are indicated by blue, green, purple, and red, respectively. **F** Neighboring evolutionary tree between GXEC-STL2 (highlight) and 25 different genera of bacteria. The phylogenetic tree was constructed on the basis of the sequences of bacterial 16S rRNA.

GXEC-STL2 formed well-defined, transparent 2-mm-diameter zones on Columbia blood agar (β-hemolysis; Figure 1A) and produced purple–black colonies with a metallic green sheen on eosin methylene blue (EMB) agar (Figure 1B). Gram staining confirmed its identity as a Gram-negative bacterium (Figure 1C). TEM revealed rod-shaped morphology (3.5 μm × 1 μm; Figure 1D). PCR amplification of virulence genes showed that GXEC-STL2 harbored the *Stx2e* gene (encoding Shiga toxin), *F18* fimbrial gene cluster, and the *irp2* gene of the high pathogenicity island (Additional file 4). The complete genome comprised 359,811 bp (Figure 1E), containing efflux pump-related genes *mdtB,* and *mdtC*, and the *yojI* gene involved in multiple drug transport. Phylogenetic analysis based on the 16S rRNA gene revealed that strain GXEC-STL2, *Escherichia marmotae*, and *Shigella dysenteriae* form a distinct and robust evolutionary clade, indicating a very close phylogenetic relationship (Figure 1F). The antimicrobial susceptibility testing results demonstrated that GXEC-STL2 was an MDR bacterial strain (Additional files 5 and 6).

### Biological characterization and genomic analysis of P-GXEC-L2P5

**Figure 2:**
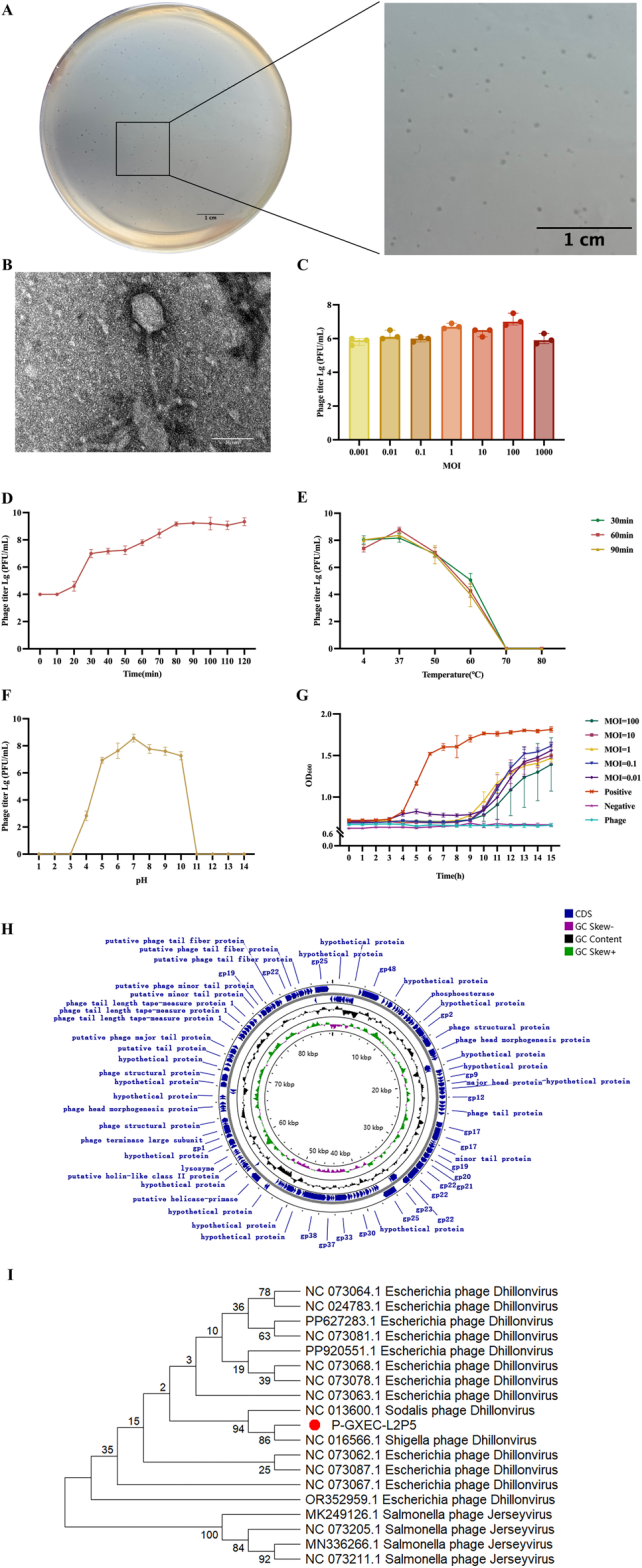
**Biological characterization and genomic analysis of P-GXEC-L2P5. A** Plaques on a double-layer agar plate. Scale bar, 1 cm. **B** Transmission electron micrograph. Scale bar, 50 nm. **C** Optimal multiplicity of infection. **D** One-step growth curve. **E** Stability at different temperatures from 4 to 80 °C. **F** Stability at various pH levels from 1 to 14. **G** In vitro bacterial inhibition by different MOI. **H** Comparative circular genome map. CDS and positive and negative GC are indicated by blue, green, and purple, respectively. **I** Neighboring evolutionary tree between P-GXEC-L2P5 and 18 different genera of phages. The phylogenetic tree was constructed on the basis of the sequences of the DNA polymerase I.

P-GXEC-L2P5 formed clear plaques with a diameter of 0.2–0.3 mm on double-layer agar plates (Figure 2A). The host spectrum of P-GXEC-L2P5 was tested against 60 strains, of which 58 were *E. coli* strains, and P-GXEC-L2P5 could infect 18 of the 58 tested *E. coli* strains (Additional file 7). TEM imaging revealed a typical regular polyhedral head (30 ± 2 nm) with an 80 ± 2-nm long tail (Figure 2B). At an MOI of 100, phage P-GXEC-L2P5 efficiency of plating was the largest and achieved maximum titer (10⁹ PFU/mL) (Figure 2C). A one-step growth curve revealed that the latent periods of P-GXEC-L2P5 were approximately 10 min (Figure 2D). For the thermal stability test, P-GXEC-L2P5 showed maintained infectivity at 4–60℃ for 90 min, with complete inactivation at 70 ℃ after 30 min (Figure 2E). The activity of P-GXEC-L2P5 remained stable at pH 5.0–10.0 but was abolished at extreme pH (pH < 5.0 or pH > 10.0) (Figure 2F). The in vitro antibacterial assay results demonstrated that P-GXEC-L2P5 exhibited the strongest inhibitory effect on host bacteria at an MOI of 100 within 9 h (Figure 2G). The genome length of phage P-GXEC-L2P5 (PQ843332.1) was 88,607 bp. Genome annotation of P-GXEC-L2P5 predicted 144 coding sequences (CDSs), including 13 DNA replication and regulation genes, 16 packaging genes, 49 structural and transporter genes, and 1 lysin gene. There were no toxins, virulence genes, or antibiotic resistance genes in the genome of phage P-GXEC-L2P5 (Figure 2H). According to the International Committee on Taxonomy of Viruses (ICTV), P-GXEC-L2P5 was classified into the genus *Dhillonvirus* within the class *Caudoviricetes*. (Figure 2I).

### P-GXEC-L2P5 prophylaxis alleviates clinical symptoms and organ lesions in GXEC-STL2-infected piglets

#### Clinical symptom scores

The clinical symptom scores of the piglets are presented in Table 1. After the bacterial challenge on day 1, both the PG and the PPG exhibited increased fecal scores and decreased vitality scores. On day 14, the PG developed obvious eyelid edema and neurological symptoms, while from day 7, all clinical parameters of the PPG remained within the normal range, consistent with those of the NG.

**Table 1:** **Clinical symptom scores**

Group	Project	Day 0	Day 1	Day 7	Day 14
Negative	Vitality	0 (0–0)	0 (0–0)	0 (0–0)	0 (0–0)
	Appetite	0 (0–0)	0 (0–0)	0 (0–0)	0 (0–0)
	Respiration	0 (0–0)	0 (0–0)	0 (0–0)	0 (0–0)
	Feces	0 (0–0)	0 (0–0)	0 (0–0)	0 (0–0)
	Eyelid edema	0 (0–0)	0 (0–0)	0 (0–0)	0 (0–0)
	Neurologic impairment	0 (0–0)	0 (0–0)	0 (0–0)	0 (0–0)
	Totals	0 (0–0)	0 (0–0)	0 (0–0)	0 (0–0)
Positive	Vitality	0 (0–0)	0 (0–1)	1 (0–1)	1 (1–2)
	Appetite	0 (0–0)	0 (0–0)	0 (0–1)	1 (0–1)
	Respiration	0 (0–0)	0 (0–0)	0 (0–0)	0 (0–0)
	Feces	0 (0–0)	0 (0–1)	0 (0–0)	0 (0–0)
	Eyelid edema	0 (0–0)	0 (0–0)	2 (1–2)	3 (2–3)
	Neurologic impairment	0 (0–0)	0 (0–0)	0 (0–0)	2 (1–2)
	Totals	0 (0–0)	1 (0–1)	3 (2–3)	6 (5–7)
Phage prophylaxis	Vitality	0 (0–0)	0 (0–1)	0 (0–0)	0 (0–0)
	Appetite	0 (0–0)	0 (0–0)	0 (0–0)	0 (0–0)
	Respiration	0 (0–0)	0 (0–0)	0 (0–0)	0 (0–0)
	Feces	0 (0–0)	1 (0–1)	0 (0–0)	0 (0–0)
	Eyelid edema	0 (0–0)	0 (0–0)	0 (0–0)	0 (0–0)
	Neurologic impairment	0 (0–0)	0 (0–0)	0 (0–0)	0 (0–0)
	Totals	0 (0–0)	1 (1–3)	0 (0–0)	0 (0–0)

The statistical analysis employed the quartile method. Data are expressed as median (first quartile, third quartile).

#### B-scan ultrasound imaging in the kidneys

**Figure 3:**
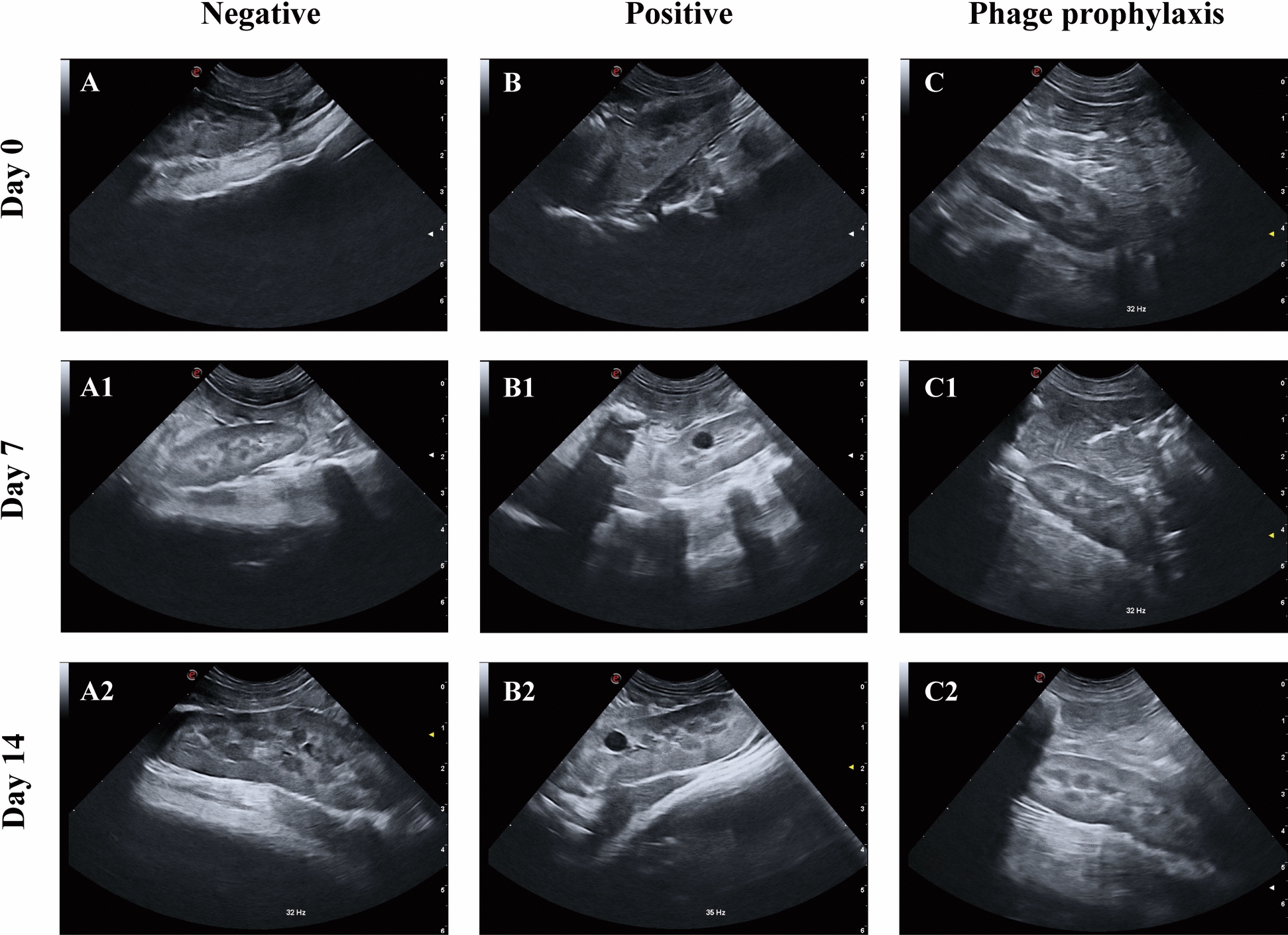
**Renal ultrasound image results in the negative group (A), the positive group (B), and the phage prophylaxis group (C) during the experiment.** No number represents day 0, the number 1 represents day 7, and the number 2 represents day 14.

Before infection, all groups exhibited kidneys with smooth contours, preserved corticomedullary differentiation, and normal sinus echogenicity, indicating healthy renal status (Figures 3A–C). After 7 days of continuous infection, the PG exhibited enhanced renal capsular echogenicity with distinct intrarenal hypoechoic areas, suggesting renal pelvis dilation (Figure 3B1), while the PPG and the NG maintained well-defined renal boundaries and preserved parenchymal architecture (Figures 3A1, C1). On day 14, persistent hypoechoic areas with concomitant cortical hyperechogenicity were observed in the kidneys of the PG (Figure 3B2), whereas the PPG and the NG exhibited no significant abnormalities in renal imaging findings (Figures 3A2, C2).

#### Gross lesions

**Figure 4:**
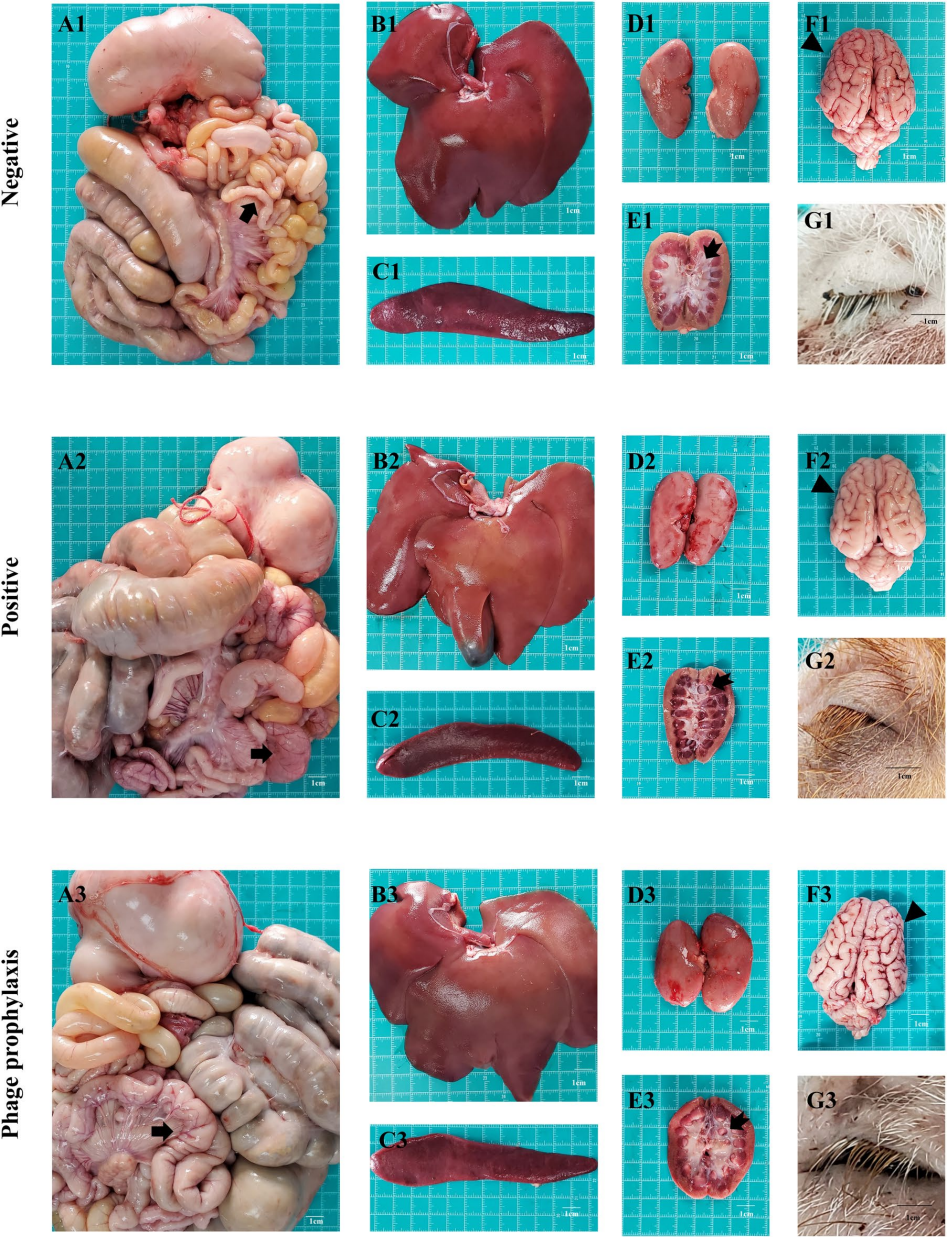
**Organ lesions of piglets.** The gross lesions of gastrointestinal tracts (**A1**, **A2**, **A3**), livers (**B1**, **B2**, **B3**), spleens (**C1**, **C2**, **C3**), kidneys (**D1**, **D2**, **D3**, **E1**, **E2**, **E3**), brains (**F1**, **F2**, **F3**), and eyelids (**G1**, **G2**, **G3**). The number 1 represents the negative group, the number 2 represents the positive group, and the number 3 represents the phage prophylaxis group. The arrows at A1, A2, and A3 designate comparative evaluation of intestinal hyperemia. The arrows at E1, E2, and E3 mark comparative analysis of renal sinus morphology. The arrows at F1, F2, and F3 indicate comparative assessment of cerebral cortical edema.

Necropsy findings revealed no gross lesions in the liver, spleen, or eyelids across all experimental groups (Figures 4B1, B2, C1, C2, G1,G2). Compared with the PPG, the PG exhibited more severe intestinal wall congestion (Figure 4A2), renal sinus space dilation (Figure 4E2), and cerebral cortical swelling (Figure 4F2). In contrast, necropsy findings in the PPG closely resembled those of the NG, with only mild congestive lesions being observed in intestinal tissues (Figures 4A1, A3), and no significant pathological alterations were detected in other organ systems (Figures 4D1, D3; E1, E3; F1, F3).

#### Histopathological changes

**Figure 5:**
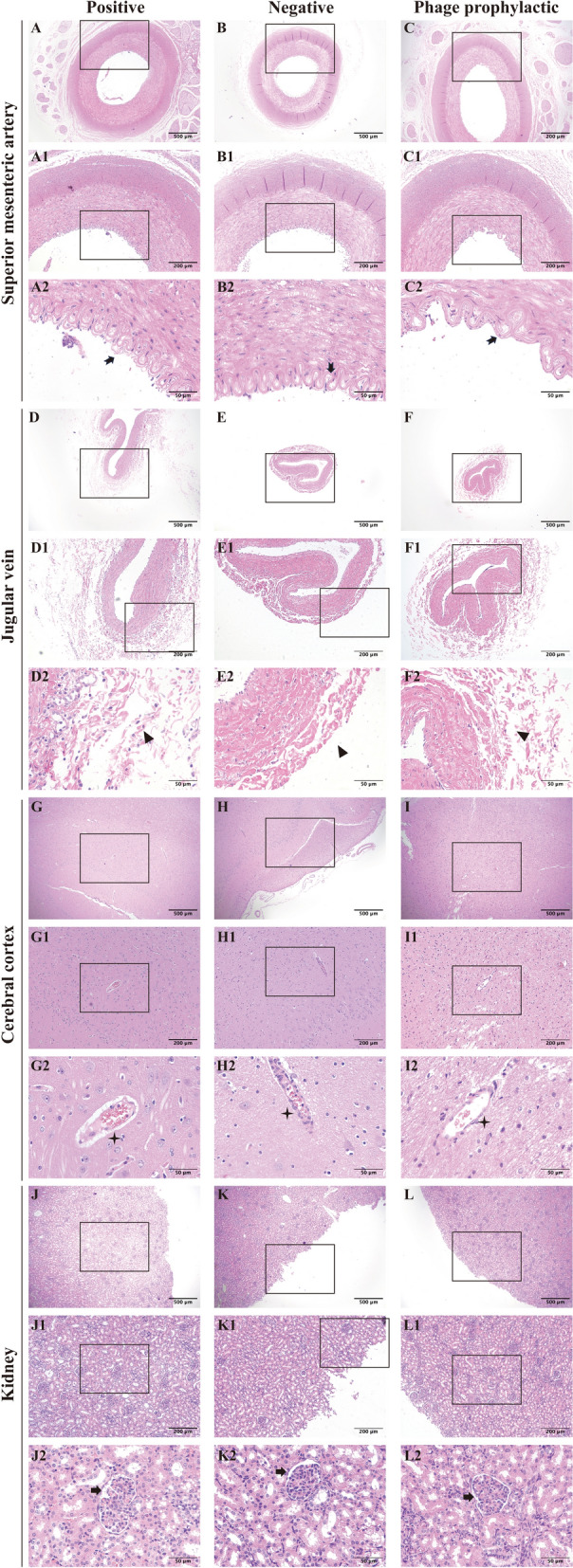
**The histopathology changes in the superior mesenteric artery (A–C), jugular vein (D–F), cerebral cortex (G–), and kidney (J–L).** No number represents 40×, scale bar, 500 μm; the number 1 represents 100×, scale bar, 200 μm; the number 2 represents 400×, scale bar, 50 μm. The arrows at A2, B2, and C2 designate the intima of the superior mesenteric artery. The arrows at D2, E2, and F2 mark the adventitia of the jugular vein. The arrows at G2, H2, and I2 indicate blood vessels in the cerebral cortex. The arrows at J2, K2, and L2 identify the Bowman’s capsule.

**Figure 6:**
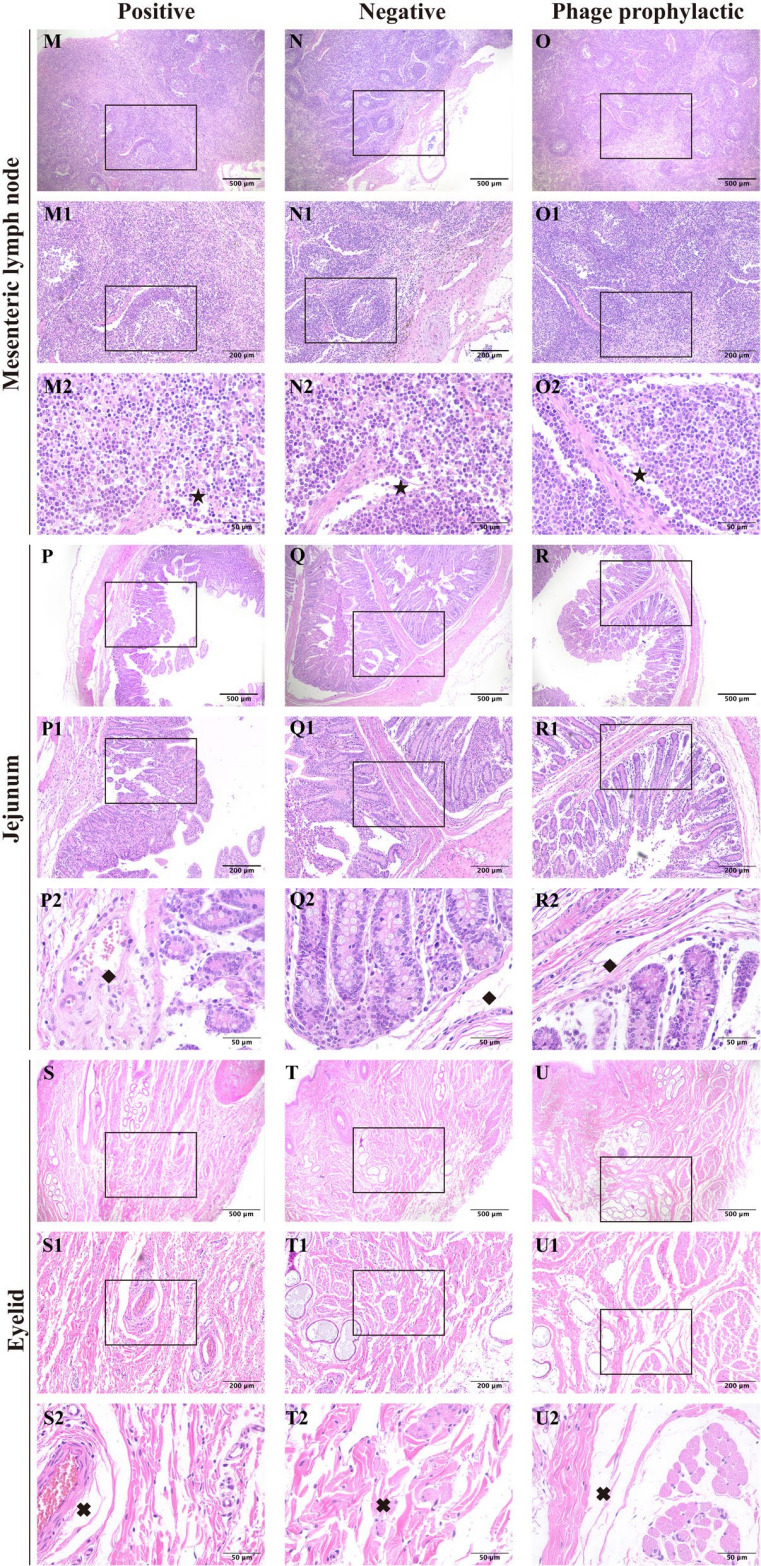
**The histopathology changes in the mesenteric lymph node (M–O), jejunum (P–R), and eyelid (S–U).** No number represents 40×, scale bar, 500 μm; the number 1 represents 50×, scale bar, 100 μm; the number 2 represents 400×, scale bar, 50 μm. The arrows at M2, N2, and O2 indicate the medullary sinuses of the mesenteric lymph nodes. The arrows at P2, Q2, and R2 point to the submucosa of the jejunum. The arrows at S2, T2, and U2 designate the dermis of the eyelid.

After GXEC-STL2 infection, the PG exhibited enlarged spaces between endothelial cells and the internal elastic lamina of the superior mesenteric artery, accompanied by structural discontinuity of the vascular intima and partial endothelial cell detachment (Figures 5A–A2). Furthermore, the jugular vein demonstrated disorganized adventitial matrix architecture and diminished cell density in the circular smooth muscle layer (Figures 5D–D2). Cerebral cortical sections revealed markedly expanded perivascular spaces with severe vacuolation of intercellular compartments, endothelial cell swelling, and indistinct basement membrane boundaries (Figures 5G–G2). Renal specimens displayed incomplete parietal epithelial cells of Bowman’s capsule, exfoliated cells within the capsular space, prominent hemorrhage in the visceral layer, and tubular epithelial cell swelling with loss of cellular demarcation (Figures 5J–J2). Mesenteric lymph nodes showed narrowed medullary sinuses containing increased lymphocyte populations (Figures 6M–M2). Jejunal tissues exhibited villous denudation, loosely arranged submucosal connective tissue, and vascular engorgement (Figures 6P–P2). In addition, the eyelid dermis showed a more dispersed interstitial matrix architecture within the loose connective tissue (Figures 6S–S2). The PPG demonstrated continuous integrity of both the intima and internal elastic lamina in the superior mesenteric artery compared with the PG (Figures 5C–C2), with the jugular vein exhibiting well-organized adventitial architecture and distinct structural integrity in the circular smooth muscle layer (Figure 5F–F2). Cerebral cortical specimens showed normal perivascular spacing, no significant vacuolation in intercellular spaces, tightly aligned endothelial cells, and uniformly thick basement membranes with sharply defined borders (Figures 5I–I2). Renal analysis revealed preserved structural clarity in both parietal and visceral layers of Bowman’s capsule, along with renal tubular epithelial cells displaying normal morphology and well-defined cytoarchitecture (Figures 5L–L2). Mesenteric lymph nodes presented widened medullary sinuses lacking lymphocyte accumulation (Figures 6O–O2), while jejunal tissues maintained intact mucosal villi, and the submucosal connective tissue was more compact without vascular congestion (Figures 6R–R2). Furthermore, the eyelid dermis exhibited enhanced cohesion within its loose connective tissue matrix (Figures 6U–U2). Collectively, these histopathological findings in the PPG closely paralleled those observed in the NG (Figures 5 B–B2, E–E2, H–H2, K–K2 and 6 N–N2, Q–Q2, T–T2).

### P-GXEC-L2P5 prophylaxis reduces STEC load, Stx2e levels, and Gb4 mRNA expression in GXEC-STL2-infected piglets

**Figure 7:**
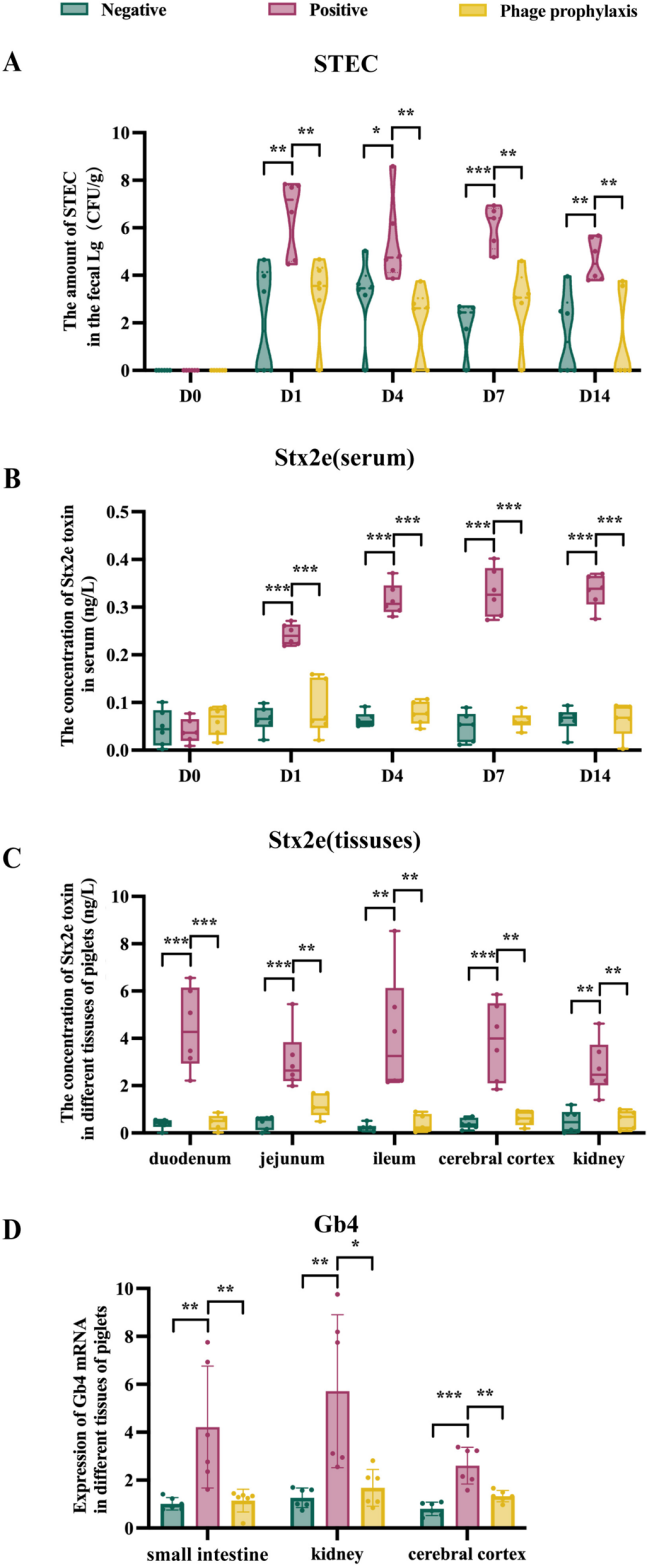
**STEC, Stx2e toxin, and receptor Gb4 assays.** **A** STEC load in the feces from day 0 to day 14. **B** Serum Stx2e level from day 0 to day 14. **C** Stx2e levels in the duodenum, jejunum, ileum, cerebral cortex, and kidney on day 14. **D** The mRNA expression of the receptor Gb4 in the intestinal tenue, kidney, and cerebral cortex on day 14. The data are presented as the means. ^*^P < 0.05, ^**^P < 0.01, and ^***^P < 0.001.

Fecal STEC load was quantified by establishing a standard curve using TaqMan probe-based qPCR and bacterial suspensions of known concentrations. The STEC colonization was not detected in any of the groups before the start of the experiment. After GXEC-STL2 infection, STEC loads were detected in feces in all groups (Figure 7A). Although the STEC load in PG feces showed an overall downward trend throughout the experimental period, it remained consistently higher (*P* < 0.05) compared with the PPG and the NG (Figure 7A). Notably, there was no significant difference in STEC load between the PPG and the NG feces from day 1 to day 14 (Figure 7A).

To further investigate the protective efficacy of phage prophylaxis, serum and tissue concentrations of Stx2e were quantified using a double-antibody sandwich enzyme-linked immunosorbent assay (ELISA). On day 0, serum Stx2e levels were very low in all groups with no significant differences (Figure 7B). After STEC infection, Stx2e concentrations in the PPG and the NG serum demonstrated negligible changes throughout the experiment, but Stx2e concentrations in the PG serum exhibited a marked elevation (*P* < 0.001), and generally showed an upward trend from day 1 to day 14 (Figure 7B). In addition, Stx2e concentrations in duodenum, jejunum, ileum, cerebral cortex, and kidney tissues were significantly higher in the PG than in the PPG and the NG (*P* < 0.01). Notably, peak concentrations were detected in duodenal tissue, whereas the lowest levels occurred in renal tissue (Figure 7C). Both the PPG and the NG showed minimal Stx2e concentrations across examined tissues, with no significant difference between the two groups (Figure 7C).

Furthermore, we analyzed the mRNA expression of Stx2e receptor Gb4 by qPCR in tissues with post-mortem lesions, including the small intestine, kidney, and cerebral cortex. The results showed that Gb4 mRNA expression in the PPG was significantly lower than that in the PG (*P* < 0.05) but not significantly different from that in the NG in all tissues examined. Notably, the PG exhibited the highest Gb4 mRNA expression in the kidney and the lowest in the cerebral cortex (Figure 7D).

### P-GXEC-L2P5 prophylaxis mitigates vascular structural damage and intercellular junction disruption in GXEC-STL2-infected piglets

#### FITC-WGA staining of vascular endothelium

**Figure 8:**
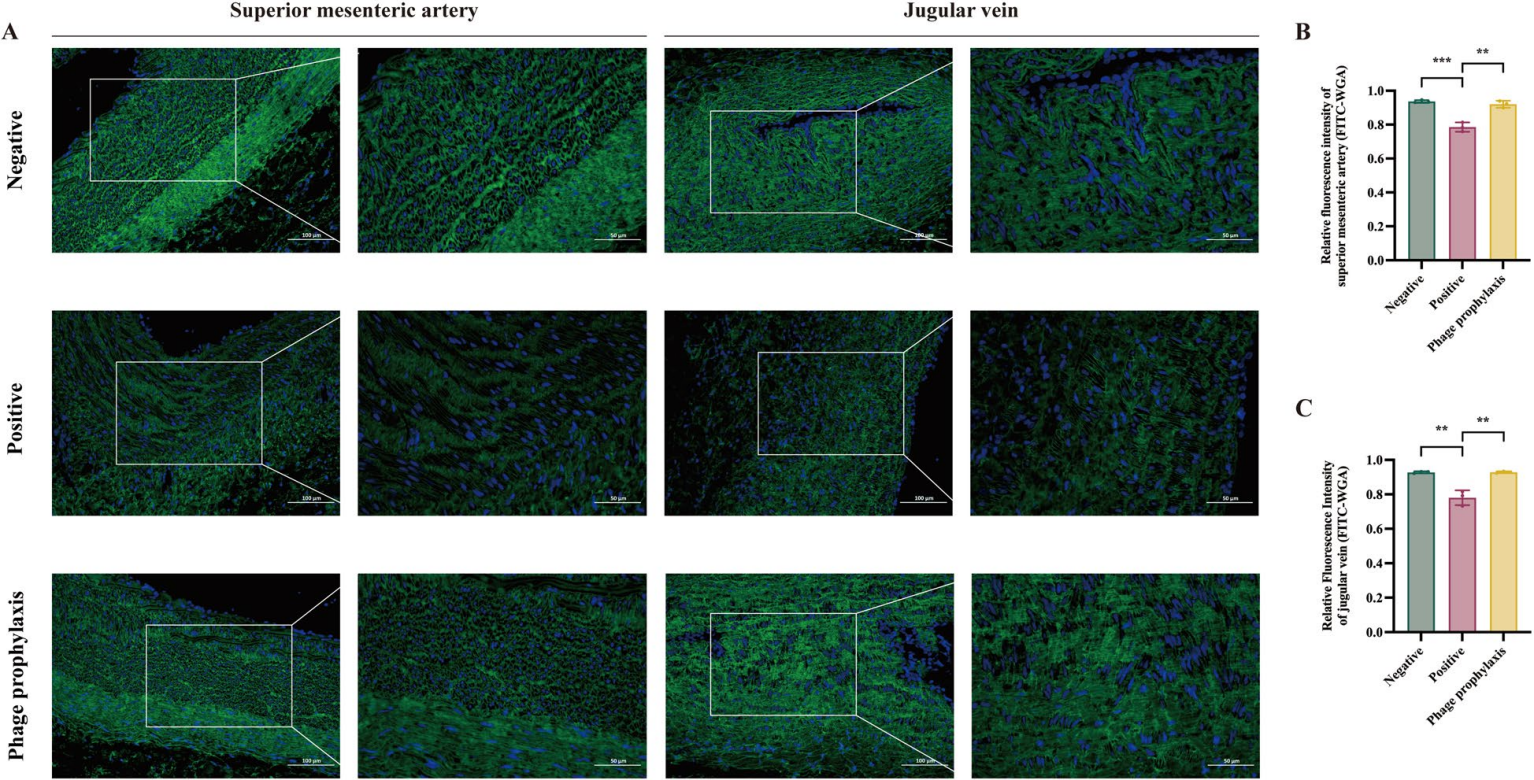
**The expression of vascular adhesive plaque proteins.** **A** FITC-WGA staining of the superior mesenteric artery and jugular vein. DAPI labels cell nuclei blue, while FITC-WGA binds to cell surface glycoproteins, imparting green fluorescence. **B** Mean fluorescence intensity of superior mesenteric artery sections. **C** Mean fluorescence intensity of jugular vein sections. ^*^P < 0.05, ^**^P < 0.01, and ^***^P < 0.001.

FITC-WGA staining was employed to evaluate the expression of adhesion proteins. The PPG and the NG exhibited tightly aligned junctions in vascular endothelial cells of the superior mesenteric artery and jugular vein. In contrast, the PG exhibited diminished green fluorescence intensity with disrupted intercellular junctions showing visible gaps (Figure 8A). The PPG demonstrated highly significant elevation in fluorescence intensity values (*P* < 0.01) for both the superior mesenteric artery and jugular vein compared with the PG, while showing no statistically significant differences compared with the NG (Figures 8B, C).

#### Quantitative analysis of mRNA expression levels for vascular endothelial integrity-associated factors

**Figure 9:**
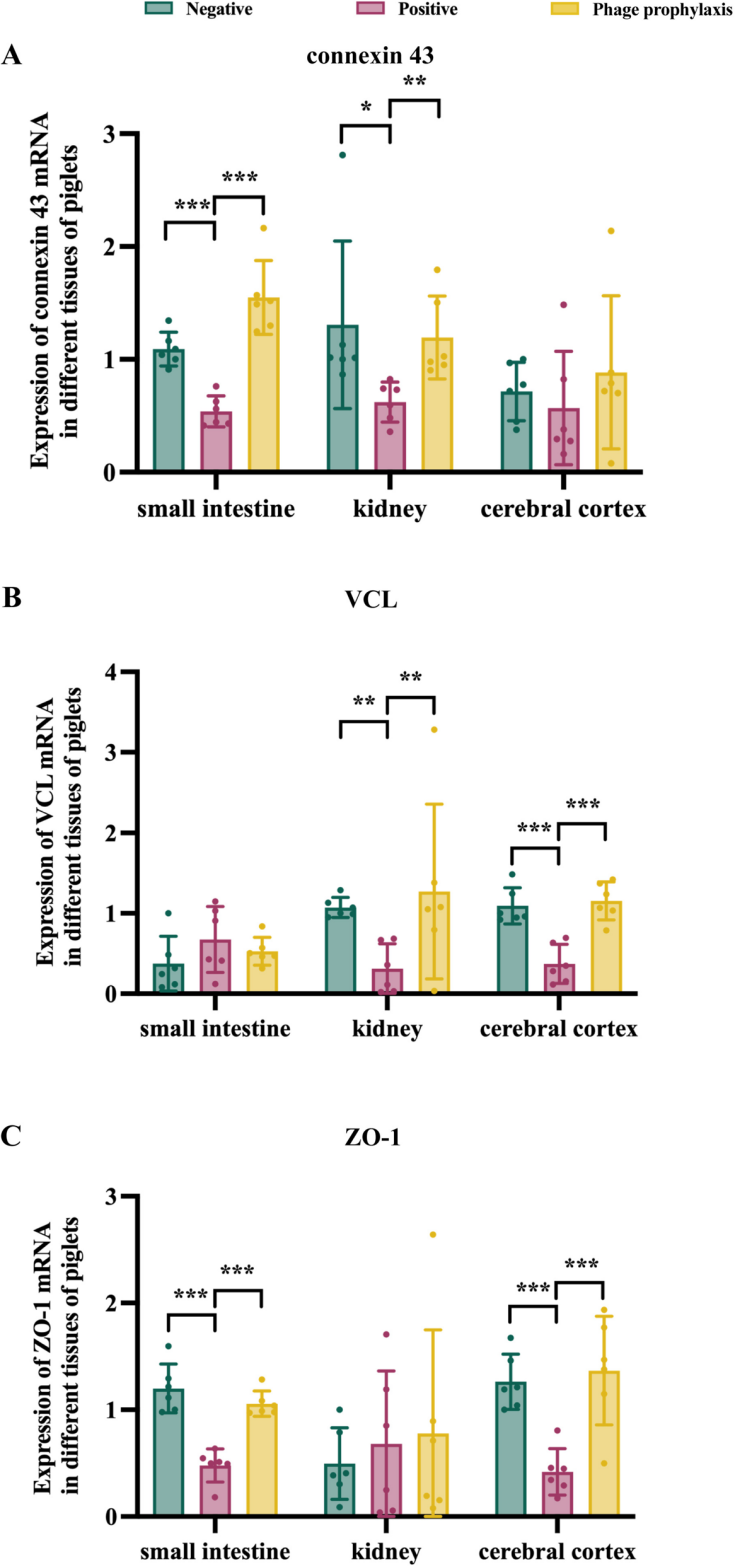
**The qPCR analysis of Cx43 (A), VCL (B), and ZO-1 (C) in the kidney, cerebral cortex, and small intestine.** The data are presented as the means. ^*^P < 0.05, ^**^P < 0.01, and ^***^P < 0.001.

To further investigate the mechanisms underlying vascular endothelial integrity impairment, subsequently, qPCR analysis was performed to quantify mRNA expression levels of gap junction protein Cx43, focal adhesion protein VCL, and tight junction protein ZO-1 in renal, cerebral cortical, and small intestinal tissues. The results demonstrated that the PPG exhibited significantly higher mRNA expression levels of Cx43 in the small intestine (*P* < 0.001) and kidney (*P* < 0.01) (Figure 9A), VCL in the kidney (*P* < 0.01) and cerebral cortex (*P* < 0.001) (Figure 9B), and ZO-1 in the small intestine (*P* < 0.001) and cerebral cortex (*P* < 0.001) (Figure 9C) when compared with the PG, with no significant difference in these mRNA expression levels from the NG.

### P-GXEC-L2P5 prophylaxis maintains the diversity of the jejunal microbiota in GXEC-STL2-infected piglets

**Figure 10:**
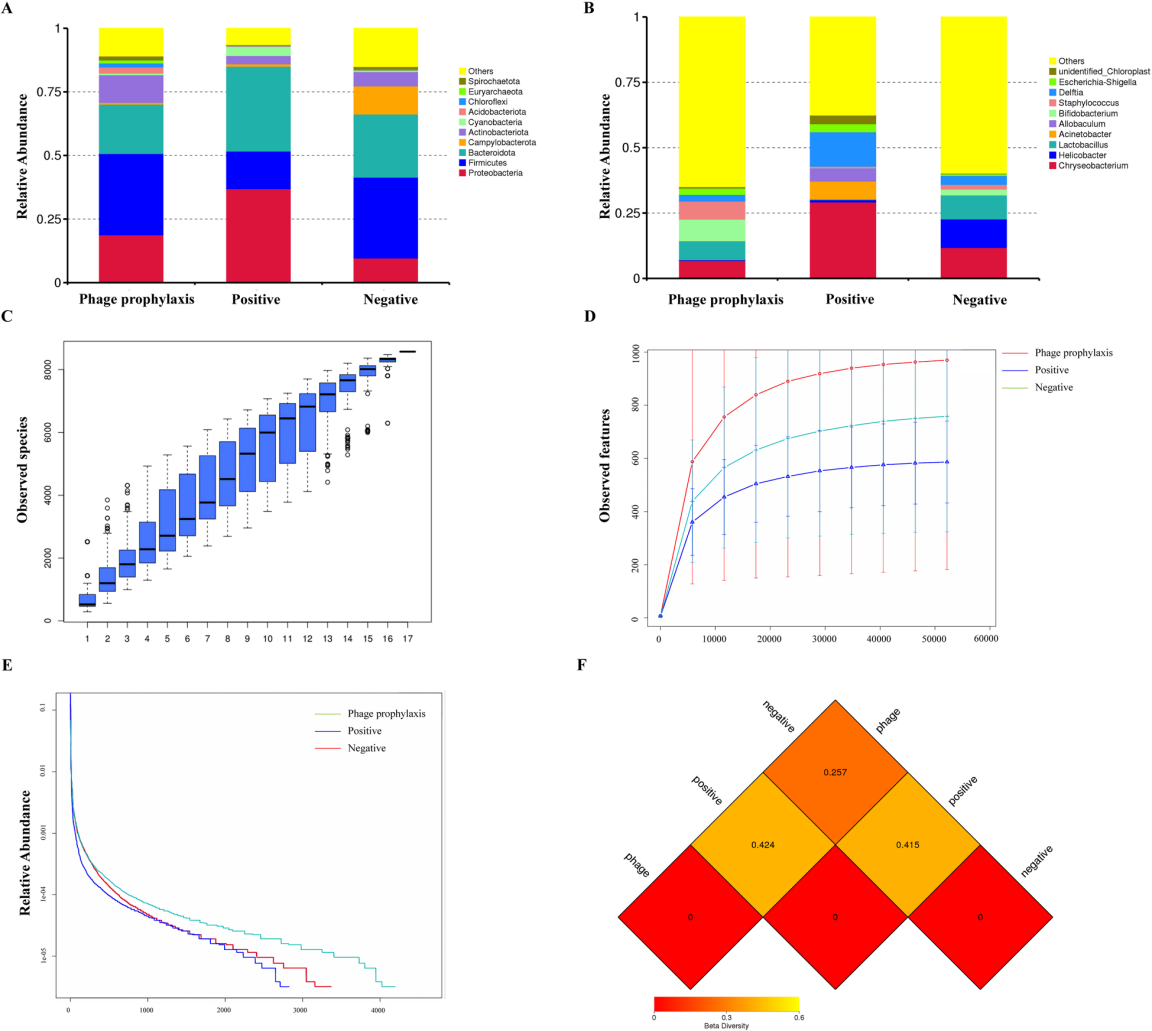
**Diversity analysis of jejunal microbiota of piglets. A** Histogram of relative species abundance at the phylum level. **B** Histogram of relative species abundance at the genus level. **C** Species accumulation box plot in jejunal samples from piglets. **D** Rarefaction curve. **E** Rank-abundance distribution curve. **F** The weighted UniFrac distance matrix heat map.

At the phylum level, the bacterial sequences from the NG samples were predominantly composed of the phyla *Firmicutes* (31.79%), the phyla *Bacteroidota* (24.81%), the phyla *Campylobacterota* (11.06%), the phyla *Proteobacteria* (9.66%), the phyla *Actinobacteriota* (5.63%), and other phyla that collectively comprised 17.05% of the total sequences (Figure 10A). The PG was largely dominated by the phylum *Proteobacteria* (36.92%), the phylum *Bacteroidota* (33.32%), the phylum *Firmicutes* (14.78%), the phylum *Cyanobacteria* (3.56%), the phylum *Actinobacteriota* (3.28%), and other phyla, which collectively accounted for 8.14% of the total sequences (Figure 10A). In the PPG, *Firmicutes* (31.93%), *Bacteroidota* (19.39%), *Proteobacteria* (18.81%), *Actinobacteriota* (10.83%), and *Acidobacteriota* (2.44%) were the predominant phyla, while the remaining phyla accounted for 16.6% of the total sequences (Figure 10A). At the genus level, the relative abundance of *Lactobacillus* decreased from an average of 9.06% in the NG to 0.27% in the PG and 7.20% in the PPG (Figure 10B), while that of *Bifidobacterium* also decreased from an average of 2.16% in the NG to 0.13% in the PG, but increased to 8.18% in the PPG (Figure 10B). Furthermore, the PG had a significantly higher relative abundance of *Acinetobacter* (0.89%) and *Delftia* (13.18%) compared with the PPG (0.16%, 2.57%) and the NG (0.19%, 3.37%) (Figure 10B). The species accumulation box plot gradually increased and eventually plateaued, indicating sufficient sample size (Figure 10C). The rarefaction curves of all groups tended to flatten, demonstrating that the sequencing data volume was progressively reasonable. Among the groups, the PPG exhibited the highest species richness, followed by the NG and the PG (Figure 10D). This was consistent with the ranking order observed in the rank-abundance curves (Figure 10E); specifically, a wider span of the curve along the horizontal axis indicates higher species abundance, while a flatter curve reflects a more uniform species distribution. As shown in Table 2, compared with the PG, the PPG had significantly higher diversity indices (Shannon, Simpson, and Chao1), while it maintained microbial diversity levels comparable to the NG. In addition, the PG had the smallest Pielou’s evenness index, which indicated the lowest community evenness—suggesting that certain species might have dominated the community. In contrast, the PPG had a higher Pielou’s evenness index than the PG, indicating the highest community evenness, which corresponded to the results in Figure 10E. The Dominance index essentially measures the consistency of the internal data within samples or the probability of sequence recurrence. The highest Dominance index in the PG meant the highest probability of sequence overlap among its samples, which also indirectly reflected its lowest species richness. The heat map visualization based on weighted UniFrac distances (Figure 10F) revealed that the PPG and the NG exhibited the smallest differences in species diversity between them. When combined with the Wilcoxon rank-sum test, significant species differences were detected between the NG–PG and PPG–PG pairs. Compared with the PG, both the PPG and the NG showed higher microbial community evenness and species diversity (Table 3).

**Table 2:** **Alpha diversity index analysis**

Group	Coverage	Observed	Chao1	Shannon	Simpson	Pieloue	Dominance
Negative	0.999	3362	3465.555	6.573	0.948	0.561	0.052
Positive	0.999	2829	2877.48	5.317	0.881	0.464	0.119
Phage prophylaxis	0.999	4194	4313.004	7.952	0.979	0.661	0.021

Coverage: reflects sampling completeness, with higher values indicating greater sequencing depth or sample representativeness; Observed: the actual number of distinct species directly identified in a sample; Chao1: estimates the total number of species present in a community sample; Shannon: integrates species richness and their relative abundance to characterize overall diversity; Simpson: measures species diversity and evenness by assessing the probability of interspecific encounters within a community; Pielou: quantifies the uniformity of species abundance distribution within a community; Dominance: represents the probability that two randomly selected sequences originate from the same sample.

**Table 3:** **Heatmap of the Wilcox test plotted in weighted UniFrac distance**

Group	MD	*P* value	Significance	LCL	UCL
NG–PPG	-1.133	0.7771		-9.18515	6.918479
NG–PG	13.467	0.0005	^***^	6.264907	20.66843
PPG–PG	14.6	0.0008	^***^	6.548188	22.65181

MD: mean difference, LCL: lower confidence limit, UCL: upper confidence limit, *** *P* ＜ 0.001

## Discussion

*Escherichia coli* is a Gram-negative bacterium that is naturally part of the intestinal microbiota. However, certain pathogenic strains of *E. coli* are associated with pathologies in the animal production industry, and STEC is one of them [5, 23]. This infection occurs in porcine farms and frequently affects nursing and weaning pigs [24]. With the formal enforcement of Regulation (EU) 2019/6 (relating to veterinary medicines) and Regulation (EU) 2019/4 (relating to medicated feed)—which strictly restrict the routine use of antimicrobials in clinical settings—the Chinese Ministry of Agriculture and Rural Affairs also issued a special rectification plan in March 2025 to standardize the use of antimicrobial veterinary drugs. These policies have collectively posed significant therapeutic challenges to the swine industry, driving the need for alternative interventions. Accordingly, phage-based strategies have emerged as critical alternatives for controlling enteric diseases in swine herds. In the present study, we isolated an MDR STEC strain and its cognate lytic phage, P-GXEC-L2P5, and subsequently employed a porcine infection model to systematically evaluate the protective efficacy of phage prophylaxis.

STEC infection in piglets typically manifests as severe clinical signs (e.g., eyelid edema and neurological dysfunction) and multiorgan pathological damage [25]. In this study, the clinical parameters of the PG showed a trend of progressive deterioration, while piglets pretreated with phage P-GXEC-L2P5 exhibited rapid recovery after STEC infection, which was similar to that of the NG. This timely resolution of clinical symptoms underscores P-GXEC-L2P5’s ability to interrupt the early progression of STEC infection.

Intestinal colonization is a prerequisite for STEC to produce Stx2e and cause systemic disease. Stx2e, in turn, is the primary virulence factor responsible for STEC-related edema disease in piglets, as it can bind to the Gb4 receptor on vascular endothelial cells, thereby inducing cell death and vascular barrier dysfunction [1, 2, 26]. In this study, we found that phage treatment significantly reduced fecal STEC load (*P* < 0.05) with a concurrent reduction in serum and tissue Stx2e toxin levels (*P* < 0.01). This reduction in bacterial load is consistent with previous studies on porcine *E. coli* phages: Mao et al. [14] reported that following treatment with phage A221, the percentage of *Enterobacteriaceae* in the duodenum of piglets decreased to 0.64%, while Li et al. [15] showed that phage cocktails lowered *E. coli O157:H7* and *Salmonella typhimurium* colonization in the jejunum. The reduction in Stx2e likely stems from two complementary mechanisms: first, P-GXEC-L2P5’s lytic activity reduces STEC load, directly decreasing toxin-producing bacteria; second, phage-induced bacterial lysis may release preformed toxins but these are likely neutralized by the host immune system or cleared before reaching target organs [27]. This is supported by the absence of toxin-mediated lesions in PPG piglets, even as phages lyse STEC in the intestine. Notably, a positive correlation was observed between Stx2e concentrations and receptor Gb4 mRNA expression. Phage administration downregulated Gb4 expression in the small intestine, kidneys, and cerebral cortex (*P* < 0.05). Few studies have investigated the effects of phages on Stx2e receptor expression. Previous work has shown that STEC infection upregulates Gb3 expression via inflammatory signaling (e.g., NF-κB activation), increasing endothelial cell susceptibility to Shiga toxins [28]. Our results suggest that P-GXEC-L2P5 may indirectly downregulate Gb4 by reducing STEC-induced inflammation—consistent with the absence of vascular lesions in PPG piglets. Alternatively, phage-derived factors may directly modulate Gb4 transcription, though this requires further investigation. Regardless, the reduction in Gb4 provides an additional layer of protection: even if low levels of Stx2e persist, fewer receptors are available for toxin binding, minimizing tissue damage.

Furthermore, via FITC-WGA staining, we demonstrated that phage pretreatment significantly enhanced vascular endothelial fluorescence intensity. This observation is particularly meaningful because the superior mesenteric artery’s endothelial integrity directly impacts intestinal barrier function—disruption here would exacerbate intestinal inflammation and toxin translocation, while preservation in PPG suggests phages limit STEC’s ability to compromise gut-vascular crosstalk. To dissect the molecular mechanisms underlying this protection, we quantified mRNA expression of three key vascular integrity-associated factors: gap junction protein Cx43, focal adhesion protein VCL, and tight junction protein ZO-1. Cx43 mediates intercellular communication via gap junctions, ensuring coordinated responses to vascular stress [29]; VCL anchors endothelial cells to the extracellular matrix, preventing detachment upon toxin exposure [30]; and ZO-1 is a scaffolding protein that forms the core of tight junctions, directly regulating vascular permeability [31]. The results showed that the PPG exhibited significantly higher mRNA levels of Cx43, VCL, and ZO-1 compared with the PG, with no differences from the NG. This indicates that P-GXEC-L2P5 plays a positive role in maintaining the transcriptional regulation of these junction proteins—likely either by reducing Stx2e load (as observed in our previous toxin quantification) or by restricting inflammatory signaling that suppresses junction protein expression, which requires further investigation.

Gut microbiota dysbiosis is critically implicated in the infection of STEC [32]. The 16S rRNA sequencing analysis revealed that STEC infection induced a significant reduction in jejunal microbial diversity (as evidenced by decreased Shannon and Simpson indices), concurrent with elevated *Proteobacteria* relative abundance, coupled with reduced *Firmicutes* levels. Phage intervention effectively preserved microbial composition, restoring beneficial genera such as *Lactobacillus* and *Bifidobacterium* to near-normal levels while suppressing overproliferation of opportunistic pathogens, including *Acinetobacter* and *Delftia*. These findings align with reports by Choi et al. [33] on phage-supplemented feed modulating gut microbiota, suggesting that phage-mediated preservation of intestinal ecological homeostasis likely reduces enteric toxin absorption and subsequent systemic inflammatory risks.

Phage therapy has emerged as a promising strategy for combating bacterial infections, owing to its unique lytic mechanisms and life cycle [34]. Despite ongoing controversies, such as narrow host specificity (limiting efficacy against diverse bacterial strains), potential horizontal gene transfer (posing theoretical risks of antibiotic resistance gene dissemination), and challenges in standardized quality control [35–37], a wealth of studies over the past decade have solidified its efficacy [14, 15, 38, 39]. Currently, commercial phage preparations find applications in human clinical therapy [7, 8], food processing [40], and broiler farming [41], among other areas. However, the development and commercialization of swine-specific phage products remain severely limited. To date, most swine-related phage research remains in the preclinical stage (i.e., laboratory-based characterization and small-scale animal trials). There is a conspicuous absence of commercially available, veterinary-licensed swine-specific phage products in major markets, including China, the European Union, and the USA. This gap is particularly problematic given the high burden of bacterial diseases in swine production. Against this backdrop, notably, the phage P-GXEC-L2P5 used in this study exhibits characteristics that address key bottlenecks in swine phage product development. Although an increase in bacterial optical density (OD) was observed at 10 h post incubation in in vitro bacteriostatic assays, this late-stage OD increase is a common phenomenon in in vitro phage-bacteria cocultures, which may be attributable to the emergence of phage-resistant bacteria. Potential mechanisms underlying resistance could include mutations in bacterial surface receptors (e.g., lipopolysaccharide or outer membrane proteins) that prevent phage adsorption or activation of the CRISPR–Cas system to target and degrade phage DNA [42]. Importantly, this in vitro resistance did not compromise the in vivo efficacy of P-GXEC-L2P5. This discrepancy may reflect the complex in vivo microenvironment, where factors such as intestinal microbiota competition, host immune clearance of resistant subpopulations, and continuous phage replication collectively suppress the expansion of resistant bacteria [43]. Furthermore, its short latent period (10 min) enables rapid bacterial lysis. Meanwhile, its broad environmental tolerance (pH 5–10, temperature 4–60 ℃) allows it to withstand harsh conditions encountered during production and gastrointestinal transit—a major limitation of many previously reported phages, which often require complex microencapsulation to maintain activity. In addition, the significant therapeutic efficacy achieved via a simple oral gavage method (phage mixed with RM) adds practical value for swine production. This administration route is easy to implement on farms and aligns with the feeding habits of weaned piglets. The emulsifying properties of RM are likely to form a protective microenvironment around phage particles, shielding them from gastric acid degradation and extending their retention time in the intestinal tract [44]—thereby enhancing their ability to target and lyse intestinal-resident STEC. This finding not only validates the feasibility of oral phage delivery in swine but also provides a low-cost, scalable strategy for future product formulation, reducing barriers to commercialization.

## Conclusions

In summary, phage P-GXEC-L2P5 reduced Stx2e toxin levels in infected piglets by decreasing STEC load, thereby mitigating Stx2e-induced vascular endothelial damage and tissue edema. As a safe and ecofriendly alternative to antibiotics, P-GXEC-L2P5 shows potential for preventing STEC-induced edema disease in piglets. These findings provide valuable insights for controlling STEC infections.

## Supplementary Information

**Additional file 1** **Virulence genes primer information.**

**Additional file 2** **Clinical symptom scoring criteria.**

**Additional file 3** **The qPCR primers and reaction conditions for vascular endothelial integrity-associated factors.**

**Additional file 4** **PCR amplification of virulence genes (M, DL2000 Marker; 1, *E. coli*; 2, *Sta*; 3, *Stb*; 4, *Stx1*; 5, *Stx2*; 6, *Stx2e*; 7, *K88*; 8, *K99*; 9, *987p*; 10, *F18*; 11, *eaeA*; 12, *irp2*; 13, *LT*; 14, ddH2O).**

**Additional file 5** **Results of the K-B disk diffusion method for determining the susceptibility of GXEC-STL2 to antibiotics.**

**Additional file 6** **Results of the K-B disk diffusion method for determining the susceptibility of GXEC-STL2 to antibiotics (A1, Tildipirosin; A2, Doxycycline; A3, Cefquinaxime; A4, Kanamycin; A5, Tylenol; B1, Enrofloxacin; B2, Mucomycin; B3, Ceftiofur; B4, Amoxicillin; B5, Gamycin; C1, Tavanamycin; C2, Gentamicin; C3, Vicodin; C4, Ampicillin; C5, Streptomycin; D1, Lincomycin; D2, Neomycin; D3, Ciprofloxacin; D4, Florfenicol; D5, Oxytetracycline)**.

**Additional file 7** **Host spectrum of phage P-GXEC-L2P5 to 58 *Escherichia coli* isolates, 2 preserved *Salmonella* isolates.** Note: “+” symbolizes positive, strain was lysed; “-” symbolizes negative, strain was not lysed. The "Undetected" in *Escherichia Coli *means the strain did not belong to these serotypes (O114: K90 (B90), O126: K71 (B16), O26: K60 (B6), O157, O157:H7, O142: K86 (B), O127a: K63 (B8), O111: K58 (B4), O86: K61 (B7), O26: K60 (B6), O111:K58 (B4), O78, O139).

## Data Availability

The genome sequence data of phage P-GXEC-L2P5 have been submitted to the National Center for Biotechnology Information (NCBI) under accession no. PQ843332.1.
