# Supplementary material for: Targeting STEC-induced edema disease in weaned piglets: prophylactic oral phage P-GXEC-L2P5 attenuates bacterial colonization, toxin production, and endothelial damage
Source: Vet Res. 2025 Dec 17;57:13. doi: 10.1186/s13567-025-01683-w (PMC12822307; doi:10.1186/s13567-025-01683-w)
Supplement: Supplementary file 1 — Additional file 1 Virulence genes primer information. [file 13567_2025_1683_MOESM1_ESM.docx]

**Additional File 1** Virulence genes primer information.

| Name | Primer Sequence (5’-3’) | Temperature (℃) | Length (bp) |
| --- | --- | --- | --- |
| *E. coli.* | F-ATGCCAGTCCAGCGTTTTTGC | 58 | 238 |
|  | R-AAAGTGTGGGTCAATAATCAGGAAGTG |  |  |
| *K88* | F-GGTGATTTCAATGGTTCGGTC | 62 | 783 |
|  | R-ATTGCTACGTTCAGCGGAGCG |  |  |
| *K99* | F-TATTATCTTAGGTGGTATGG | 50 | 314 |
|  | R-GGTATCCTTTAGCAGCAGTATTTC |  |  |
| *987P* | F-CTGCCAGTCTATGCCAAGTG | 58 | 500 |
|  | R-ACGGTGTACCTGCTGAACGAATAG |  |  |
| *F18* | F-GTGAAAAGACTAGTGTTTATTTC | 50 | 510 |
|  | R-CTTGTAAGTAACCGCGTAAGC |  |  |
| *Sta* | F-GAAACAACATGACGGGAGGT | 56 | 227 |
|  | R-GCACAGGCAGGATTACAACA |  |  |
| *Stb* | F-TGCCTATGCATCTACACAATC | 55 | 216 |
|  | R-GCAGTGAGAAATGGACAATG |  |  |
| *LT* | F-CGGCGTTACTATCCTCTCTA | 55 | 378 |
|  | R-ATTGGGGGTTTTATTATTCC |  |  |
| *Stx1* | F-CGATGTTACGGTTTGTTACTGTGACAGC | 62 | 664 |
|  | R-AATGCCACGCTTCCCAGAATTG |  |  |
| *Stx2* | F-GTTTTGACCATCTTCGTCTGATTATTGAG | 62 | 281 |
|  | R-AGCGTAAGGCTTCTGCTGTGAC |  |  |
| *Stx2e* | F-AATAGTATACGGACAGCGAT | 58 | 422 |
|  | R-TCTGACATTCTGGTTGACTC |  |  |
