# Supplementary material for: Targeting STEC-induced edema disease in weaned piglets: prophylactic oral phage P-GXEC-L2P5 attenuates bacterial colonization, toxin production, and endothelial damage
Source: Vet Res. 2025 Dec 17;57:13. doi: 10.1186/s13567-025-01683-w (PMC12822307; doi:10.1186/s13567-025-01683-w)
Supplement: Supplementary file 2 — Additional file 2 Clinical symptom scoring criteria. [file 13567_2025_1683_MOESM2_ESM.docx]

**Additional File 2** Clinical symptom scoring criteria.

| Clinical symptoms | Score | | | |
| --- | --- | --- | --- | --- |
|  | 0 | 1 | 2 | 3 |
| Vitality | Good | Decreased | Bad | Dead |
| Appetite | Good | Slightly poor | Poor | Not eating |
| Respiration | Normal  （20–40 times/min） | Slightly quick  （41–60 times/min） | Quick  （＞60 times/min） | - |
| Feces | Normal | Loose stool | Moderate diarrhea | Severe diarrhea |
| Eyelid edema | Normal | Mild | Moderate | Severe |
| Neurologic impairment | Normal | Slightly | Moderate | Severe |
