# Supplementary material for: Targeting STEC-induced edema disease in weaned piglets: prophylactic oral phage P-GXEC-L2P5 attenuates bacterial colonization, toxin production, and endothelial damage
Source: Vet Res. 2025 Dec 17;57:13. doi: 10.1186/s13567-025-01683-w (PMC12822307; doi:10.1186/s13567-025-01683-w)
Supplement: Supplementary file 3 — Additional file 3 The qPCR primers and reaction conditions for vascular endothelial integrity-associated factors. [file 13567_2025_1683_MOESM3_ESM.docx]

**Additional File 3** The qPCR primers and reaction conditions for vascular endothelial integrity-associated factors.

| Name | Primer Sequence (5’-3’) | GenBank/Accession No. |
| --- | --- | --- |
| *GAPDH* | F-TGAAGGTCGGAGTGAACGGAT | 396823 |
|  | R-TGGGTGGAATCATACTGGAAC |  |
| *ZO-1* | F-CTATGTCCAGAATCTCGGAAAAG | 396567 |
|  | R-CCTTCCCCTCAGAAACCCATAC |  |
| *VCL* | F-CAATGCCCAGAATCTGATGCAA | NM_213934.1 |
|  | R-AGTGTAAATCCAGCATCCGTTC |  |
| *Cx43* | F-GAGGCGTGCCTACTTCACTT | NM_001244212.1 |
|  | R-CAGCGGTGGAATAGGCTTGA |  |
| *Gb4* | F-CCTCCTGCTGCTGTCACTCC | XM_047449137.1 |
|  | R-TCACATCTGAAGGGTGGGAG |  |
