# Supplementary figures and images for: Targeting STEC-induced edema disease in weaned piglets: prophylactic oral phage P-GXEC-L2P5 attenuates bacterial colonization, toxin production, and endothelial damage
Source: Vet Res. 2025 Dec 17;57:13. doi: 10.1186/s13567-025-01683-w (PMC12822307; doi:10.1186/s13567-025-01683-w)

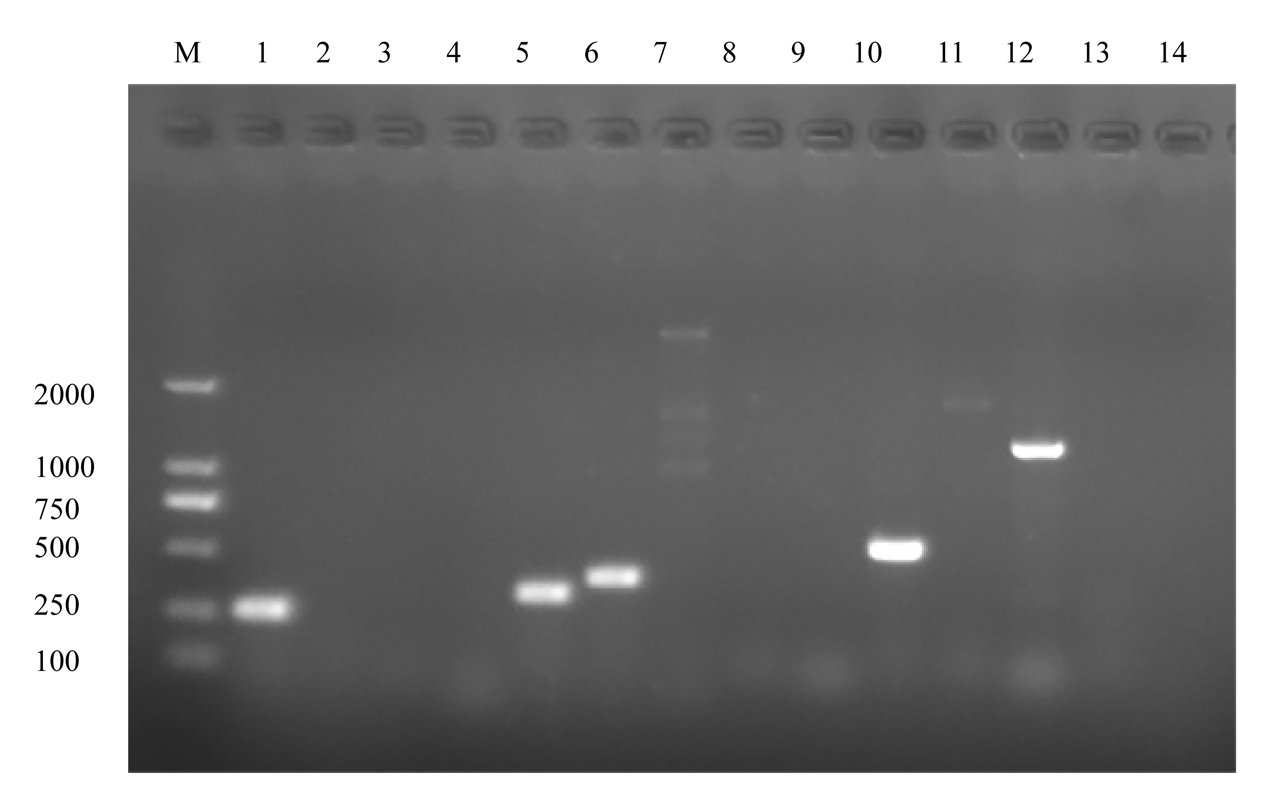

Supplement: Supplementary file 4 — Additional file 4 PCR amplification of virulence genes (M, DL2000 Marker; 1, E. coli; 2, Sta; 3, Stb; 4, Stx1; 5, Stx2; 6, Stx2e; 7, K88; 8, K99; 9, 987p; 10, F18; 11, eaeA; 12, irp2; 13, LT; 14, ddH2O). [file 13567_2025_1683_MOESM4_ESM.tif]

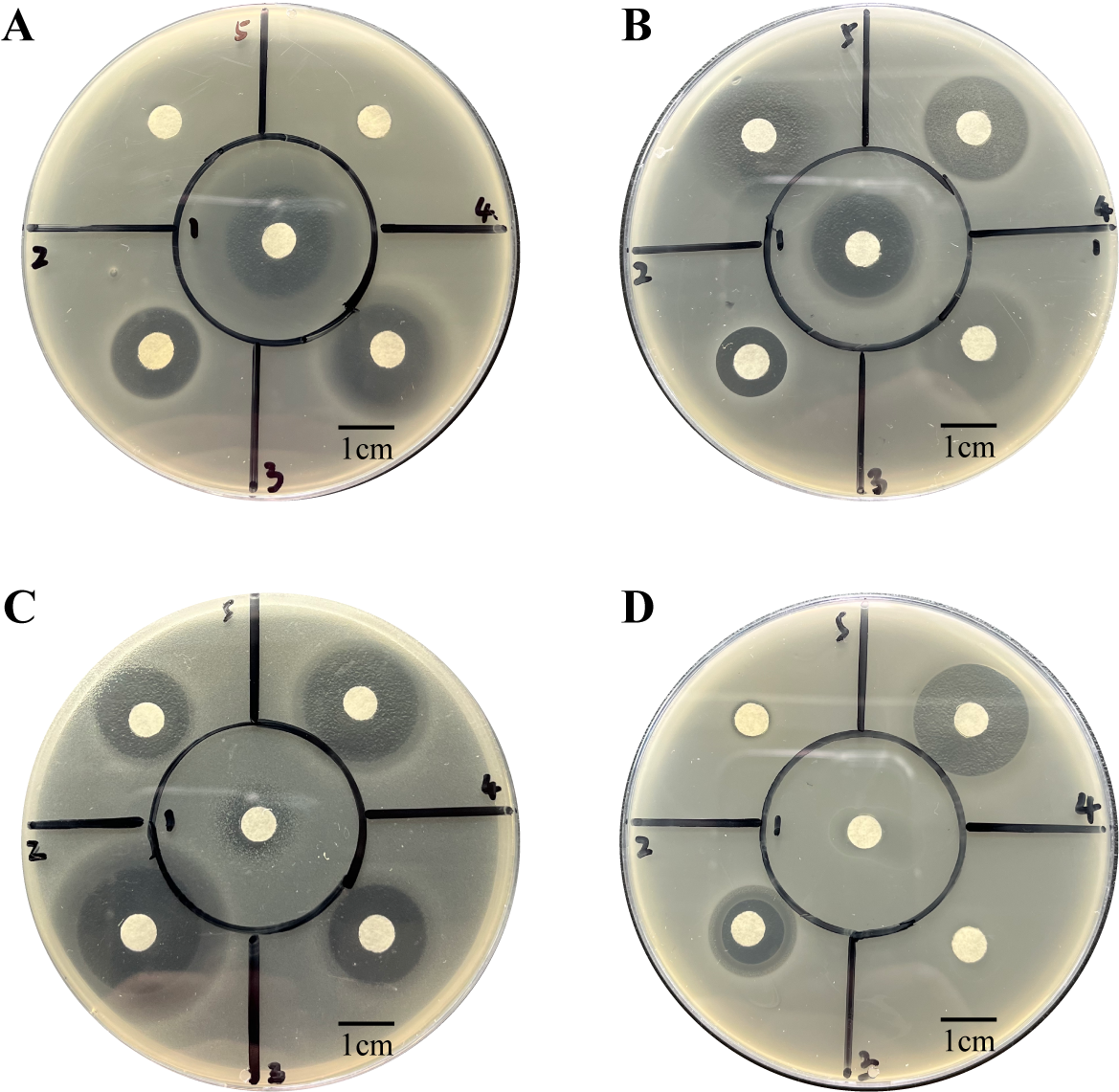

Supplement: Supplementary file 6 — Additional file 6 Results of the K-B disk diffusion method for determining the susceptibility of GXEC-STL2 to antibiotics (A1, Tildipirosin; A2, Doxycycline; A3, Cefquinaxime; A4, Kanamycin; A5, Tylenol; B1, Enrofloxacin; B2, Mucomycin; B3, Ceftiofur; B4, Amoxicillin; B5, Gamycin; C1, Tavanamycin; C2, Gentamicin; C3, Vicodin; C4, Ampicillin; C5, Streptomycin; D1, Lincomycin; D2, Neomycin; D3, Ciprofloxacin; D4, Florfenicol; D5, Oxytetracycline). [file 13567_2025_1683_MOESM6_ESM.tif]
