# Supplementary material for: Targeting STEC-induced edema disease in weaned piglets: prophylactic oral phage P-GXEC-L2P5 attenuates bacterial colonization, toxin production, and endothelial damage
Source: Vet Res. 2025 Dec 17;57:13. doi: 10.1186/s13567-025-01683-w (PMC12822307; doi:10.1186/s13567-025-01683-w)
Supplement: Supplementary file 5 — Additional file 5 Results of the K-B disk diffusion method for determining the susceptibility of GXEC-STL2 to antibiotics. [file 13567_2025_1683_MOESM5_ESM.docx]

**Additional File 5** Results of the K-B disk diffusion method for determining the susceptibility of GXEC-STL2 to antibiotics.

| Antibiotic | Criteria of bacteriostatic zone (mm) | | | Inhibition circle diameter (mm) | Tolerance |
| --- | --- | --- | --- | --- | --- |
|  | Resistance (R) | Intermediary (I) | Sensitive (S) |  |  |
| Tildipirosin | ≤12 | 13-17 | ≥18 | 18 | S |
| Doxycycline | ≤14 | 15-18 | ≥19 | 15 | I |
| Cefquinaxime | ≤18 | 19-23 | ≥24 | 16 | R |
| Kanamycin | ≤10 | 11-14 | ≥15 | 6 | R |
| Tylenol | ≤12 | 13-14 | ≥15 | 6 | R |
| Enrofloxacin | ≤12 | 13-16 | ≥17 | 17 | I |
| Mucomycin | ≤11 | 12-15 | ≥16 | 11 | R |
| Ceftiofur | ≤11 | 12-22 | ≥23 | 6 | R |
| Amoxicillin | ≤13 | 14-17 | ≥18 | 19 | S |
| Gamycin | ≤15 | 16-19 | ≥20 | 14 | R |
| Tavanamycin | ≤12 | 13-15 | ≥16 | 12 | R |
| Gentamicin | ≤12 | 13-14 | ≥15 | 20 | S |
| Vicodin | ≤15 | 16-19 | ≥20 | 14 | R |
| Ampicillin | ≤7 | 8-11 | ≥12 | 20 | S |
| Streptomycin | ≤10 | 11-13 | ≥14 | 14 | S |
| Lincomycin | ≤10 | 11-15 | ≥16 | 6 | R |
| Neomycin | ≤12 | 13-15 | ≥16 | 11 | R |
| Ciprofloxacin | ≤15 | 16-20 | ≥21 | 6 | R |
| Florfenicol | ≤12 | 13-17 | ≥18 | 20 | S |
| Oxytetracycline | ≤12 | 13-15 | ≥16 | 6 | R |
