# Supplementary material for: Targeting STEC-induced edema disease in weaned piglets: prophylactic oral phage P-GXEC-L2P5 attenuates bacterial colonization, toxin production, and endothelial damage
Source: Vet Res. 2025 Dec 17;57:13. doi: 10.1186/s13567-025-01683-w (PMC12822307; doi:10.1186/s13567-025-01683-w)
Supplement: Supplementary file 7 — Additional file 7 Host spectrum of phage P-GXEC-L2P5 to 58 Escherichia coli isolates, 2 preserved Salmonella isolates. Note: “+” symbolizes positive, strain was lysed; “-” symbolizes negative, strain was not lysed. The "Undetected" in Escherichia Coli means the strain did not belong to these serotypes (O114: K90 (B90), O126: K71 (B16), O26: K60 (B6), O157, O157:H7, O142: K86 (B), O127a: K63 (B8), O111: K58 (B4), O86: K61 (B7), O26: K60 (B6), O111:K58 (B4), O78, O139). [file 13567_2025_1683_MOESM7_ESM.docx]

**Additional File 7** Host spectrum of phage P-GXEC-L2P5 to 58 *Escherichia coli* isolates, 2 preserved *Salmonella* isolates.

| NO. | Strains | Genus | Place | Source | Serotype | Lysis |
| --- | --- | --- | --- | --- | --- | --- |
| 1 | GXEC-N01 | *Escherichia Coli* | Guangxi | Porcine | O114：K90 (B90) | **-** |
| 2 | GXEC-N02 | *Escherichia Coli* | Guangxi | Human | Undetected | **-** |
| 3 | GXEC-N03 | *Escherichia Coli* | Guangxi | Human | Undetected | **+** |
| 4 | GXEC-N04 | *Escherichia Coli* | Guangxi | Porcine | O126: K71 (B16) | **+** |
| 5 | GXEC-N05 | *Escherichia Coli* | Guangxi | Porcine | O26: K60 (B6) | **+** |
| 6 | GXEC-N06 | *Escherichia Coli* | Guangxi | Porcine | O126: K71 (B16) | **-** |
| 7 | GXEC-N07 | *Escherichia Coli* | Guangxi | Porcine | O157 | **-** |
| 8 | GXEC-N08 | *Escherichia Coli* | Guangxi | Porcine | Undetected | **+** |
| 9 | GXEC-N09 | *Escherichia Coli* | Guangxi | Porcine | Undetected | **-** |
| 10 | GXEC-N10 | *Escherichia Coli* | Guangxi | Porcine | Undetected | **-** |
| 11 | GXEC-N11 | *Escherichia Coli* | Guangxi | Porcine | O142: K86 (B） | **-** |
| 12 | GXEC-N12 | *Escherichia Coli* | Guangxi | Human | Undetected | - |
| 13 | GXEC-N13 | *Escherichia Coli* | Guangxi | Porcine | Undetected | - |
| 14 | GXEC-N14 | *Escherichia Coli* | Guangxi | Porcine | Undetected | - |
| 15 | GXEC-N15 | *Escherichia Coli* | Guangxi | Porcine | Undetected | - |
| 16 | GXEC-N16 | *Escherichia Coli* | Guangxi | Porcine | Undetected | - |
| 17 | GXEC-N17 | *Escherichia Coli* | Guangxi | Porcine | Undetected | - |
| 18 | GXEC-N18 | *Escherichia Coli* | Guangxi | Porcine | Undetected | - |
| 19 | GXEC-N19 | *Escherichia Coli* | Guangxi | Porcine | Undetected | - |
| 20 | GXEC-N20 | *Escherichia Coli* | Guangxi | Porcine | Undetected | - |
| 21 | GXEC-N21 | *Escherichia Coli* | Guangxi | Porcine | Undetected | - |
| 22 | GXEC-N22 | *Escherichia Coli* | Guangxi | Porcine | Undetected | - |
| 23 | GXEC-N23 | *Escherichia Coli* | Guangxi | Porcine | Undetected | - |
| 24 | GXEC-N24 | *Escherichia Coli* | Guangxi | Porcine | Undetected | - |
| 25 | GXEC-N25 | *Escherichia Coli* | Guangxi | Porcine | Undetected | - |
| 26 | GXEC-N26 | *Escherichia Coli* | Guangxi | Porcine | Undetected | - |
| 27 | GXEC-N27 | *Escherichia Coli* | Guangxi | Human | Undetected | - |
| 28 | GXEC-B01 | *Escherichia Coli* | Guangxi | Pet | Undetected | **+** |
| 29 | GXEC-B02 | *Escherichia Coli* | Guangxi | Pet | Undetected | - |
| 30 | GXEC-C01 | *Escherichia Coli* | Guangxi | Bovine | Undetected | - |
| 31 | GDEC-F01 | *Escherichia Coli* | Guangdong | Porcine | Undetected | **+** |
| 32 | GDEC-F02 | *Escherichia Coli* | Guangdong | Porcine | Undetected | **+** |
| 33 | GDEC-F03 | *Escherichia Coli* | Guangdong | Environment | Undetected | **+** |
| 34 | GDEC-F04 | *Escherichia Coli* | Guangdong | Environment | O127a: K63 (B8) | **-** |
| 35 | GDEC-F05 | *Escherichia Coli* | Guangdong | Environment | O127a: K63 (B8) | **-** |
| 36 | GDEC-F06 | *Escherichia Coli* | Guangdong | Environment | O127a: K63 (B8) | **-** |
| 37 | GDEC-F07 | *Escherichia Coli* | Guangdong | Environment | O111: K58 (B4) | **+** |
| 38 | GDEC-F08 | *Escherichia Coli* | Guangdong | Environment | Undetected | **-** |
| 39 | GDEC-F09 | *Escherichia Coli* | Guangdong | Environment | Undetected | - |
| 40 | GDEC-F10 | *Escherichia Coli* | Guangdong | Environment | Undetected | - |
| 41 | GDEC-F11 | *Escherichia Coli* | Guangdong | Raw pork | O86: K61 (B7) | **+** |
| 42 | GDEC-F12 | *Escherichia Coli* | Guangdong | Raw pork | Undetected | **+** |
| 43 | GDEC-F13 | *Escherichia Coli* | Guangdong | Porcine | O26: K60 (B6) | **+** |
| 44 | GDEC-F14 | *Escherichia Coli* | Guangdong | Raw pork | Undetected | - |
| 45 | GDEC-F15 | *Escherichia Coli* | Guangdong | Environment | Undetected | - |
| 46 | SCEC-Z01 | *Escherichia Coli* | Sichuan | Avian | Undetected | - |
| 47 | SCEC-Z02 | *Escherichia Coli* | Sichuan | Avian | O111：K58 (B4) | - |
| 48 | SCEC-Z03 | *Escherichia Coli* | Sichuan | Avian | Undetected | **+** |
| 49 | SCEC-Z04 | *Escherichia Coli* | Sichuan | Avian | Undetected | **+** |
| 50 | SCEC-Z05 | *Escherichia Coli* | Sichuan | Avian | Undetected | **+** |
| 51 | SCEC-Z06 | *Escherichia Coli* | Sichuan | Avian | Undetected | - |
| 52 | SCEC-Z07 | *Escherichia Coli* | Sichuan | Avian | Undetected | - |
| 53 | SCEC-Z08 | *Escherichia Coli* | Sichuan | Avian | Undetected | - |
| 54 | ATCC25922 | *Escherichia Coli* | ATCC |  | Undetected | - |
| 55 | CVCC4050 | *Escherichia Coli* | CVCC |  | O157:H7 | - |
| 56 | GXEC-STL1 | *Escherichia Coli* | Guangxi | Porcine | O78 | **+** |
| 57 | GXEC-STL2 | *Escherichia Coli* | Guangxi | Porcine | O139 | **+** |
| 58 | GXEC-STC1 | *Escherichia Coli* | Guangxi | Porcine | Undetected | **+** |
| 59 | CVCC1806 | *Salmonella typhimurium* | CVCC | Avian | S. Enteritidis | - |
| 60 | CVCC3384 | *Salmonella typhimurium* | CVCC | Porcine | S. Typhimurium | - |

Note: “+” symbolizes positive, strain was lysed; “-” symbolizes negative, strain was not lysed.

The "Undetected" in *Escherichia Coli* means the strain did not belong to these serotypes (O114: K90 (B90), O126: K71 (B16), O26: K60 (B6), O157, O157:H7, O142: K86 (B), O127a: K63 (B8), O111: K58 (B4), O86: K61 (B7), O26: K60 (B6), O111:K58 (B4), O78, O139)
